# Magnetic Interplay between *π*‐Electrons of Open‐Shell Porphyrins and *d*‐Electrons of Their Central Transition Metal Ions

**DOI:** 10.1002/advs.202105906

**Published:** 2022-03-18

**Authors:** Qiang Sun, Luis M. Mateo, Roberto Robles, Pascal Ruffieux, Giovanni Bottari, Tomás Torres, Roman Fasel, Nicolás Lorente

**Affiliations:** ^1^ nanotech@surfaces Laboratory Empa ‐ Swiss Federal Laboratories for Materials Science and Technology Dübendorf 8600 Switzerland; ^2^ Materials Genome Institute Shanghai University Shanghai 200444 China; ^3^ Departamento de Química Orgánica Universidad Autónoma de Madrid Madrid 28049 Spain; ^4^ IMDEA‐Nanociencia Campus de Cantoblanco Madrid 28049 Spain; ^5^ Centro de Física de Materiales CFM/MPC (CSIC‐UPV/EHU) Paseo de Manuel de Lardizabal 5 Donostia‐San Sebastián 20018 Spain; ^6^ Institute for Advanced Research in Chemical Sciences (IAdChem) Universidad Autónoma de Madrid Madrid 28049 Spain; ^7^ Department of Chemistry Biochemistry and Pharmaceutical Sciences University of Bern Bern 3012 Switzerland; ^8^ Donostia International Physics Center (DIPC) Donostia‐San Sebastián 20018 Spain

**Keywords:** density functional theory calculations, molecular magnetism, on‐surface synthesis, organic open‐shell compounds, porphyrins

## Abstract

Magnetism is typically associated with *d*‐ or *f*‐block elements, but can also appear in organic molecules with unpaired *π*‐electrons. This has considerably boosted the interest in such organic materials with large potential for spintronics and quantum applications. While several materials showing either *d*/*f* or *π*‐electron magnetism have been synthesized, the combination of both features within the same structure has only scarcely been reported. Open‐shell porphyrins (Pors) incorporating *d*‐block transition metal ions represent an ideal platform for the realization of such architectures. Herein, the preparation of a series of open‐shell, *π*‐extended Pors that contain magnetically active metal ions (i.e., Cu^II^, Co^II^, and Fe^II^) through a combination of in‐solution and on‐surface synthesis is reported. A detailed study of the magnetic interplay between *π*‐ and *d*‐electrons in these metalloPors has been performed by scanning probe methods and density functional theory calculations. For the Cu and FePors, ferromagnetically coupled *π*‐electrons are determined to be delocalized over the Por edges. For the CoPor, the authors find a Kondo resonance resulting from the singly occupied Co^II^ *d*_z_^2^ orbital to dominate the magnetic fingerprint. The Fe derivative exhibits the highest magnetization of 3.67 *μ_B_* (S≈2) and an exchange coupling of 16 meV between the *π*‐electrons and the Fe *d*‐states.

## Graphical abstract

Using a combination of in‐solution and on‐surface chemistry, three *π*‐extended metallo‐porphyrins are prepared (namely, **CuPorA_2_**, **CoPorA_2_**, and **FePorA_2_**). An atomic‐scale study of these nanostructures—by scanning probe microscopy and density functional theory—reveals a magnetic interplay between the *π*‐ and *d*‐electrons. The presented approach paves the way towards the realization of fascinating novel materials with intriguing magnetic properties.

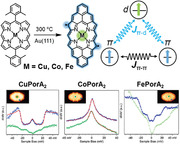

## 1 Introduction

Carbon magnetism, which originates from the presence of unpaired *π*‐electrons in organic *π*‐conjugated systems, has been the subject of extensive studies in recent years.^[^ ^1^, ^2^, ^3^, ^4^, ^5^, ^6^, ^7^, ^8^ ^]^ The remarkable properties of the carbon *π*‐electrons allow for long spin coherence times and distances, which is highly beneficial for spintronic applications.^[^ ^9^, ^10^ ^]^ In particular, large magnetic exchange couplings can be achieved in nanographenes, ensuring magnetic stability under practical room temperature conditions.^[^ ^1^, ^5^ ^]^ On the other hand, spin‐orbit coupling and the resulting magnetic anisotropy energy (MAE) associated with *d*‐ or *f*‐block elements is at the heart of conventional inorganic magnetic materials and essential for a wide spectrum of applications, including permanent magnets and information storage devices. Therefore, research on materials with a strong interplay between the *p*/*π*‐ and *d*‐electrons or orbitals is particularly interesting, and has indeed led to the discovery of exotic phenomena, such as the Zhang‐Rice singlet state (which is responsible for unconventional superconductivity in cuprates),^[^ ^11^, ^12^ ^]^ and aromaticity in conjugated metallocycles.^[^ ^13^ ^]^ However, an atomic‐scale insight into the magnetic interactions between the *π*‐ and *d*‐electrons in metal–organic structures is still missing to the best of our knowledge.

There are two main prerequisites for magnetic materials with contributions from both *π*‐ and *d*‐electrons. First, the target organic structure must present a *π*‐conjugated magnetic/open‐shell electronic structure, and second, the *π*‐system must have a sufficiently large overlap with some *d*‐orbitals, allowing for *π*‐*d* magnetic interactions. Meeting these two requirements within one molecule is certainly not straightforward, especially since the preparation of organic open‐shell compounds is typically limited by their inherently poor stability and solubility. In this context, porphyrins (Pors) are highly appealing molecular platforms thanks to their planar structure, large aromatic 18 *π*‐electron conjugated system, and structural versatility.^[^ ^14^, ^15^ ^]^ Moreover, Pors offer the possibility to chelate transition metals within their inner cavity, which potentially allows for strong *π*‐*d* electron interactions.^[^ ^16^ ^]^

Indeed, Pors have successfully been used as a scaffold for the preparation of organic *π*‐delocalized radicals.^[^ ^17^, ^18^, ^19^, ^20^, ^21^, ^22^ ^]^ This goal was mainly achieved by the use of redox chemistry and/or the fusion of Pors to polyaromatic hydrocarbons (PAHs) with inherent open‐shell character.^[^ ^23^, ^24^, ^25^, ^26^, ^27^, ^28^, ^29^, ^30^ ^]^ However, synthetic access to such Por‐based open‐shell structures often suffers from limitations, such as multi‐step synthesis, low yields, and poorly soluble intermediates and products. Furthermore, the low stability of these species, arising from their inherently low highest occupied molecular orbital (HOMO) – lowest occupied molecular orbital (LUMO) gap, typically hampers their characterization, which needs to be carried out in degassed solutions or through the in situ generation of the radical species.^[^ ^19^ ^]^

**Figure 1:**
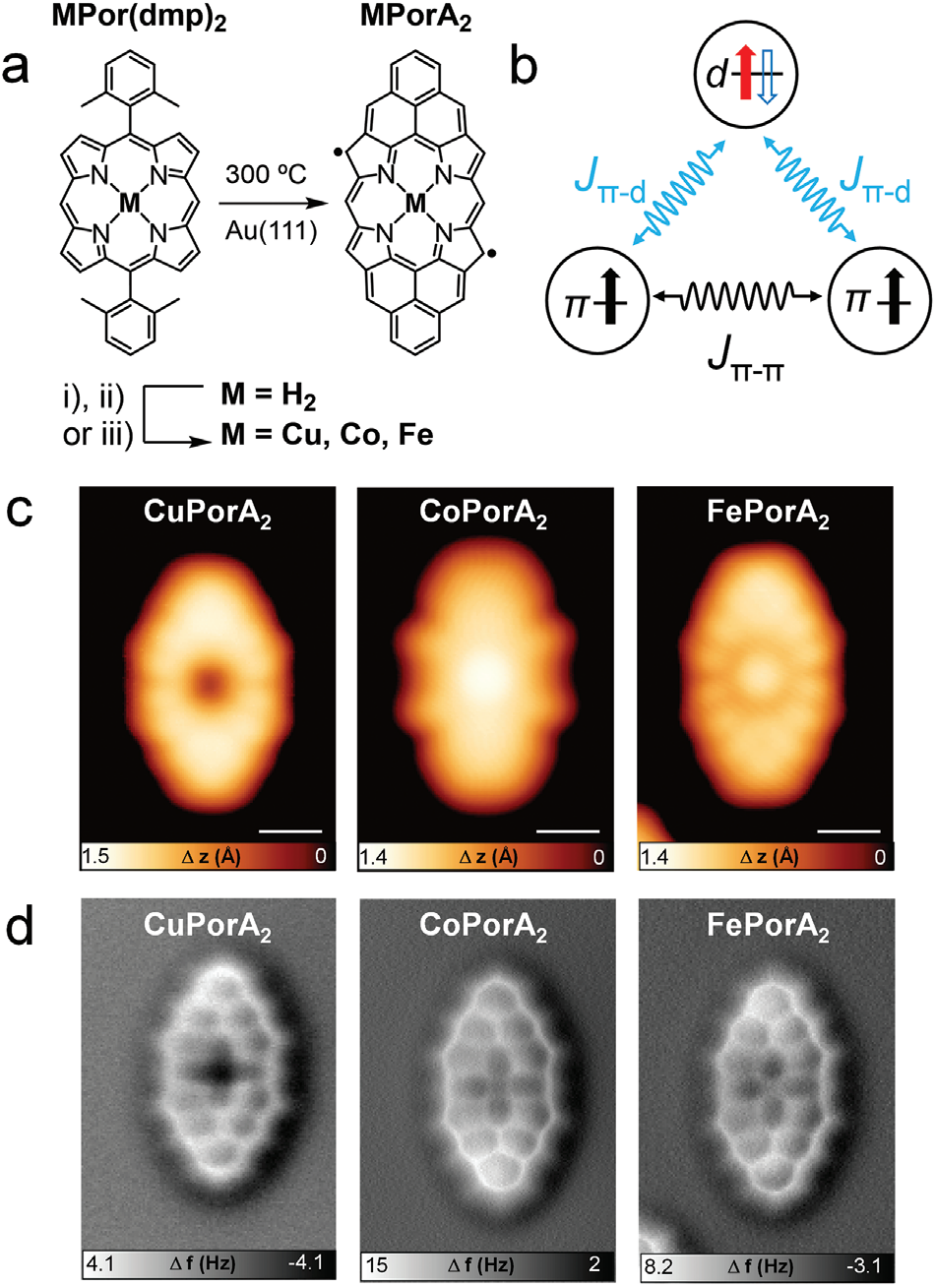
Solution‐based and on‐surface synthesis and characterization of **CuPorA_2_**, **CoPorA_2_**, and **FePorA_2_**. a) Solution‐based and on‐surface synthesis of MPorA_2_ on Au(111). Conditions: i) Cu(OAc)_2_, THF, reflux overnight for **CuPorA_2_**; ii) Co(OAc)_2_·4H_2_O, DMF, 100 °C overnight for **CoPorA_2_**; iii) FeCl_2_, DMF, 120 °C overnight for **FePorA_2_**. b) Schematic representation of the exchange coupling between the *π*‐ and *d*‐electrons. The filled black arrow indicates a singly occupied *π*‐orbital, the filled red arrow indicates a singly occupied *d*‐orbital, and the empty blue arrow indicates a singly unoccupied *d*‐orbital. c) STM and d) nc‐AFM images of copper, cobalt, and iron complexed Pors. Scanning parameters: **CuPorA_2_**: *V*_s_ = ‒0.2 V, *I*_t_ = 250 pA; **CoPorA_2_**: *V*_s_ = ‒0.06 V, *I*_t_ = 200 pA; **FePorA_2_**: *V*_s_ = ‒0.04 V, *I*_t_ = 160 pA. Scale bars: 5 Å.

Over the past decades, on‐surface synthesis has emerged as a promising tool for the preparation of open‐shell PAH systems, offering significant advantages over the more classical “wet” synthesis. The employed ultra‐high vacuum (UHV) conditions and the use of atomically clean, catalytic metal surfaces, allow for both the synthesis and characterization of such structures that are hardly accessible through solution‐based methods.^[^ ^31^, ^32^, ^33^, ^34^, ^35^, ^36^, ^37^, ^38^, ^39^, ^40^ ^]^ These tools have also repeatedly been employed for the preparation and study of Pors and related porphyrinoids,^[^ ^41^, ^42^ ^]^ focusing mainly on model systems like porphines,^[^ ^43^, ^44^ ^]^ tetraphenylPors,^[^ ^45^, ^46^, ^47^ ^]^ and derivatives thereof.^[^ ^48^, ^49^, ^50^ ^]^ Also, *π*‐extended Pors were prepared by fusing the macrocycle with PAHs,^[^ ^51^, ^52^, ^53^, ^54^ ^]^ but the focus of these studies was not laid on the possible open‐shell character of the *π*‐system. More recently, we used on‐surface synthesis to fabricate **ZnPorA_2_**, a Por bearing two *meso*‐fused phenalenyl units (**Figure** **1**a). For the nanostructure **ZnPorA_2_**, we demonstrated an open‐shell character with a ferromagnetic (*S* = 1) ground state,^[^ ^55^ ^]^ which furthermore confers a peculiar reactivity to this Por, allowing the fabrication of *meso–meso* triply fused ZnPor nanotapes (NTs).^[^ ^56^ ^]^ The magnetic properties of the open‐shell **ZnPorA_2_**, as well as the resulting Por NTs, were unequivocally identified by high‐resolution scanning probe microscopy at the single‐molecule scale.^[^ ^55^, ^56^ ^]^

In the present work, taking advantage of the diradical open‐shell nature of doubly phenalenyl‐fused Pors,^[^ ^55^ ^]^ we have incorporated three metal ions (i.e., Cu^II^, Co^II^, and Fe^II^) in the inner Por cavity and investigated the magnetic properties of the resulting metalated nanostructures (**CuPorA_2_**, **CoPorA_2_**, and **FePorA_2_**, Figure 1a). We have used scanning tunneling spectroscopy (STS) to characterize their electronic and magnetic features. The results were rationalized by comprehensive density functional theory (DFT) calculations, performed both in the gas phase and on Au(111).

## 2 Solution‐ and On‐Surface Synthesis of **CuPorA_2_**, **CoPorA_2_**, and **FePorA_2_**

The synthesis of the three studied Pors was carried out in a two‐step procedure, as shown in Figure 1a. First, a previously reported free‐base Por (**H_2_Por(dmp)_2_**)^[^ ^55^ ^]^ was reacted in solution with the corresponding metal salts Cu(OAc)_2_, Co(OAc)_2_•4H_2_O, and FeCl_2_, to obtain **CuPor(dmp)_2_**, **CoPor(dmp)_2_**, and **FePor(dmp)_2_**, respectively. After an aqueous workup, evaporation of the organic solvents, column chromatography, and size exclusion chromatography, the corresponding metalated Pors were obtained in good to excellent yields (see Section S3, Supporting Information). Subsequently, **CuPor(dmp)_2,_** **CoPor(dmp)_2_**, and **FePor(dmp)_2_** were individually sublimed onto the clean Au(111) surface under UHV conditions, followed by thermal activation at 300 °C to promote cyclodehydrogenation^[^ ^57^ ^]^ and afford fully planarized, conjugated *π*‐extended metalloPors **CuPorA_2_**, **CoPorA_2_**, and **FePorA_2_**, respectively (Figure 1a).^[^ ^55^, ^56^ ^]^ The successful formation of the three target compounds was unambiguously confirmed by high‐resolution STM and bond‐resolved nc‐AFM imaging (Figure 1c).^[^ ^58^ ^]^

## 3 Experimental Characterization of the Three Metalated Pors

As previously reported by us, **ZnPorA_2_**, with the closed‐shell *d* ^10^ metal ion, exhibits a diradical open‐shell character with two unpaired *π*‐electrons delocalized over the “edges” of the macrocycle.^[^ ^55^ ^]^ The two *π*‐electrons are ferromagnetically coupled in their ground state (i.e., triplet state) and can be excited into the singlet state with excitation energy of 19.4 mV, as determined from STS measurements.^[^ ^55^ ^]^ It can therefore be expected that the introduction of unpaired *d*‐electrons in the PorA_2_ structure, through the complexation of magnetically active ions (such as Cu^II^, Co^II^, and Fe^II^), may give rise to an exchange coupling between the metal‐centered unpaired *d*‐electron(s) and the *π*‐electrons on the Por scaffold, as schematically illustrated in Figure 1b.

The different electronic structures of the three *π*‐extended metalloPors are immediately reflected in the respective topographic STM images, which show different contrasts over the central metals. More specifically, **CuPorA_2_** exhibits a negligible density of state (DOS) in the middle of the cavity, while **CoPorA_2_** and **FePorA_2_** have prominent contributions (Figure 1c). Similar features are also found in the respective nc‐AFM images, in which **CuPorA_2_** shows less contrast over the central metal atom than **CoPorA_2_** and **FePorA_2_** (Figure 1d). DFT calculations, performed both in the gas phase and on Au(111), reveal that this is not related to different adsorption geometries of the surface‐supported molecules, but rather originates from their electronic properties (vide infra). In fact, all the transition metals lie in the same plane of the molecules (see DFT results in Figure S2.6, Supporting Information).

As previously reported in the literature and by ourselves, the unpaired electrons in magnetically active metalloPors and nanographenes are readily passivated through hydrogenation.^[^ ^8^, ^54^, ^55^, ^56^, ^59^ ^]^ Indeed, besides intact metalloPors (Figure 1c,d), hydrogenated analogs of such metalated macrocycles are also frequently observed (examples shown in Figure S2.1, Supporting Information), which reflects the inherent open‐shell character of these Pors with magnetic ions.

To address the magnetic properties of **CuPorA_2_**, **CoPorA_2_**, and **FePorA_2_**, we have carried out low‐temperature STS measurements. This technique has successfully been used for the study of a plethora of magnetically active atoms and molecules on surfaces.^[^ ^60^ ^]^ For such species, an excitation between their different magnetic states or changes of the magnetic anisotropy can result in inelastic tunneling processes (so‐called inelastic spin excitation). The latter leads to a step‐like increase in differential conductance (d*I*/d*V*) at bias threshold voltages that are symmetrically located around the Fermi level.^[^ ^61^ ^]^ In addition, the itinerant electrons from the metal substrate may interact with localized spins, leading to the observation of anomalies in the DOS in proximity to the Fermi energy (i.e., the many‐body Kondo state).^[^ ^62^, ^63^ ^]^ These features provide direct experimental evidence for the determination of the magnetic properties of the systems even in absence of an external magnetic field. Furthermore, thanks to the atomically sharp tip used for STS, the electronic and magnetic features of surface‐supported nanostructures can be mapped over the entire molecule and thus be characterized at the atomic scale. Since the energy scales of such magnetic fingerprints are typically within a few tens of meV, the d*I*/d*V* spectra were measured in the low‐bias range of ±100 mV.

**Figure 2:**
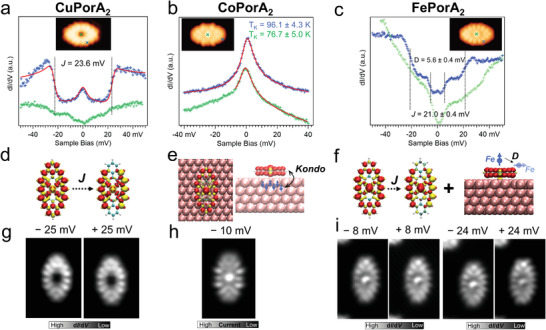
Scanning tunnelling spectroscopy measurements on **CuPorA_2_**, **CoPorA_2_**, and **FePorA_2_**. a) STS spectra of **CuPorA_2_** acquired at the positions marked by the crosses with corresponding colors in the inset STM image. The solid line is a fit based on a dynamic scattering model,^[^ ^64^ ^]^ which determines a ferromagnetic exchange coupling strength of 23.6 mV. b) STS spectra of **CoPorA_2_** acquired at the positions indicated by the crosses with corresponding colors in the inset STM image. The data are fit by a Frota function (solid red lines). A strong contribution of a Co state is seen in the STS spectrum over the Co atom, and therefore is not taken into account for the fit. The Kondo temperatures for the edge and metal are indicated (see Figure S2.3, Supporting Information, for details). c) STS spectra of **FePorA_2_** acquired at the positions indicated by the crosses with corresponding colors in the inset STM image. Two steps centered around the Fermi level are discernable for the metal while a single step is seen for the edge. d) Schematics showing the excitation of the ferromagnetic ground state to antiferromagnetic states of **CoPorA_2_**, e) the Kondo screening of **CoPorA_2_** on Au(111), f) the excitation between the ferromagnetic and antiferromagnetic states of **FePorA_2_** and the magnetic anisotropy excitation of the Fe atom. g) Constant height STS maps of **CuPorA_2_** taken at bias voltages of −25 and 25 mV (*V*_mod_ = 4 mV). h) Constant height current STM image of **CoPorA_2_** taken at −10 mV. i) Constant height STS maps of **FePorA_2_** taken at bias voltages of ± 8 and ± 24 mV, respectively (*V*_mod_ = 4 mV).

For the spectra taken on the edge of **CuPorA_2_**, we found two conductance steps in the d*I*/d*V* spectrum, symmetrically distributed around the Fermi level, as well as a peak at zero bias (**Figure** **2**a). This feature is similar to what has been observed for **ZnPorA_2_**, and a consequence of a triplet *S* = 1 ground state, where two spins are ferromagnetically coupled.^[^ ^55^ ^]^ By constructing an *S* = 1 system with two ferromagnetically coupled spins, we fit the data with a dynamic scattering model^[^ ^64^ ^]^ and extract an exchange coupling energy *J* = 23.6 meV, slightly larger than that of the **ZnPorA_2_** with 19.4 meV. Additionally, from the fit, we obtain a non‐zero Kondo scattering parameter *Jρ*_s_ of −0.11, where *J* represents the coupling strength between the magnetic impurity and the metal surface and *ρ*_s_ is the free electron density. The non‐zero Kondo scattering parameter is a result of the cusp above the excitation energy and the intensity of the zero‐bias peak in the spectrum. Note that, for **CuPorA_2_**, we do not find any characteristic features for the spectrum obtained over the central Cu atom (vide infra).

Turning to **CoPorA_2_**, we observe prominent zero‐bias resonances over both the Por edge and the central Co ion (Figure 2b). The resonance acquired on the edge can be nicely fit by the Frota function,^[^ ^65^ ^]^ and its half‐width at half maximum (HWHM) used to determine the corresponding Kondo temperature. Because of a strong molecular state localized on the Co metal ion being close to the Fermi level (peak at −0.1 V, see wide‐range d*I*/d*V* spectra in Figure S2.2, Supporting Information), the data points below −8 mV were not taken into account for the fitting of the spectra of **CoPorA_2_**. Both zero‐bias resonances (on the edge and on the metal core) exhibit characteristic temperature‐dependent broadening. By extracting the HWHM of the spectra at elevated temperatures and further fitting them with the Fermi‐liquid model (Figure S2.3, Supporting Information), we obtained similar Kondo temperatures over the edge (*T*_k_ = 96.1 K) and the central Co atom (*T*_k_ ≈76.7 K), which indicates the same origin of the Kondo resonance (see also discussion later). Besides the Kondo resonance, we do not observe other low‐bias STS features.

Finally, the STS spectra of **FePorA_2_** are shown in Figure 2c. The spectrum acquired over the edge of the molecule displays two pairs of conductance steps centered around the Fermi level, which indicate two inelastic excitations at thresholds of 5.6 ± 0.4 and 21.0 ± 0.4 mV, respectively. Meanwhile, the spectrum over the Fe ion presents a dip around the Fermi level, which can also be interpreted as two conductance steps with a threshold excitation energy of ≈5.5 meV, in accordance with one of the inelastic excitations on the edge. Fe‐based Pors can have multiple spins, originating from the unpaired *3d* electrons, and possess high magnetic moments and relatively large MAE on surfaces.^[^ ^51^, ^66^ ^]^ Regardless of the presence or absence of fused nanographenes, MAE of around a few meV have been reported for such Fe‐based Pors on Au(111).^[^ ^51^ ^]^ We therefore assign the conductance step in the spectrum taken over the Fe atom to the MAE resulting from the Fe *3d*‐electrons.

**Figure 3:**
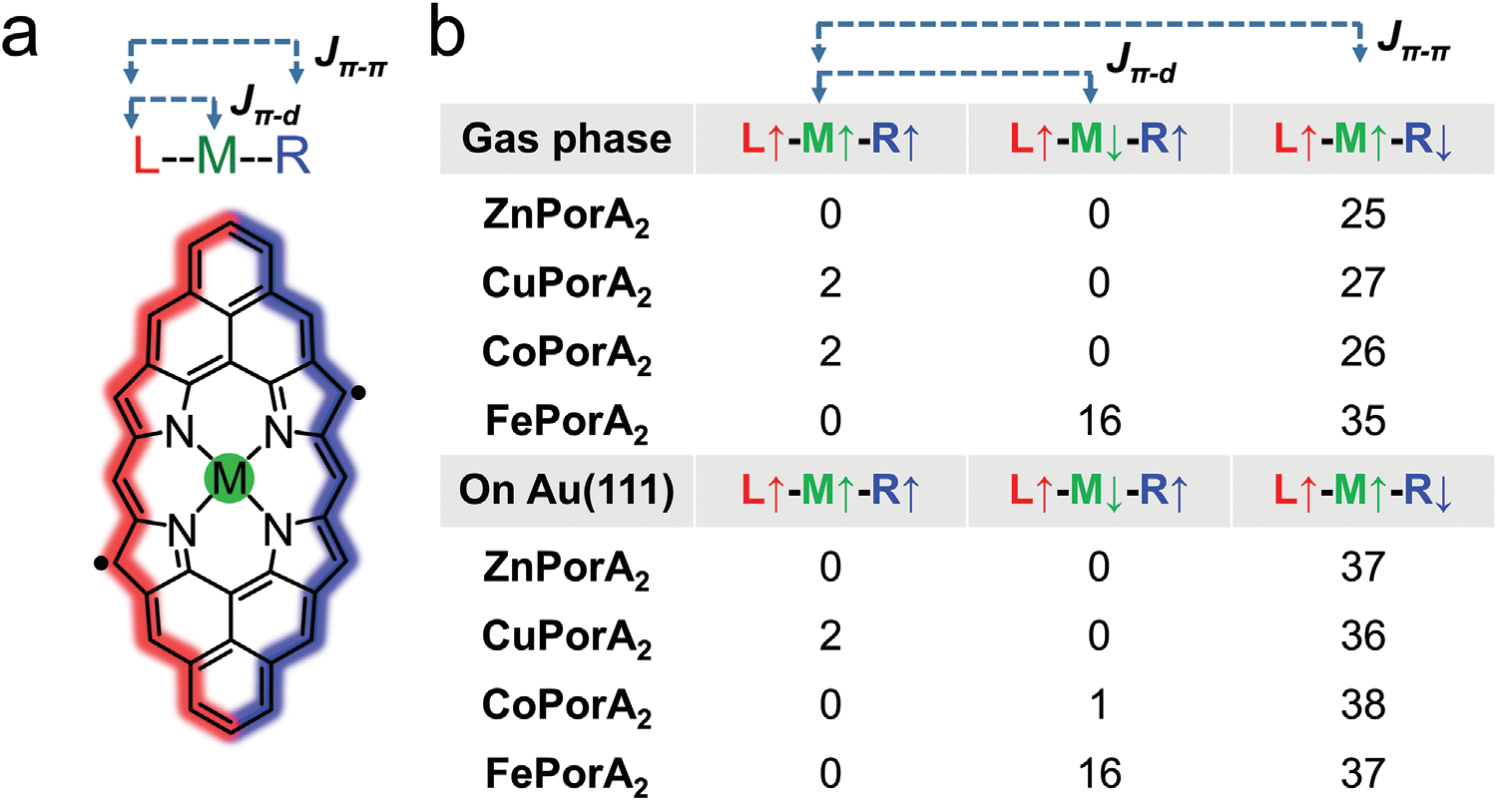
Calculations of magnetic properties of **CuPorA_2_**, **CoPorA_2_**, and **FePorA_2_**. a) Chemical model of MPorA_2_, the color contours represent the spin densities of *π‐*electrons over the left (red) and right (blue) edges. M denotes the central metal ion. b) DFT calculated relative energies of different magnetic states for MPorA_2_ in the gas phase and on Au(111). L, M, and R stand for the left part, metal ion, and right part of the corresponding molecules, respectively. The arrows indicate the polarization direction of the spin densities. Energy unit: meV.

To further understand the magnetic properties of the three investigated metalloPors and to rationalize the observed STS data, we have performed comprehensive DFT calculations. Since the Por‐complexed transition metals may carry magnetic moments, the spin density distributions within the Pors can be divided into three parts: the left edge (L), the central metal ion (M), and the right edge (R), as illustrated in **Figure** **3**a. Depending on the relative alignment of the spins in the three parts, different magnet couplings can be obtained (Figure S2.4, Supporting Information). The gas‐phase calculations show that, for the three metalloPors studied in this work, the spins at the two edges are ferromagnetically coupled in the most stable solution, i.e., *J*_*π*–*π*_ >0. This is consistent with the previously studied case of **ZnPorA_2_**,^[^ ^55^ ^]^ and the energy difference with regard to the antiferromagnetic solutions (*J*_*π*–*π*_ <0) is ≈25–35 meV (see summarized results in Figure 3b). The coupling strength between the metal ion and the edges, i.e., *J*_*π*‐d_, is weak for **CuPorA_2_** and **CoPorA_2_** (1–2 meV), and significantly stronger for **FePorA_2_** (16 meV).

**Figure 4:**
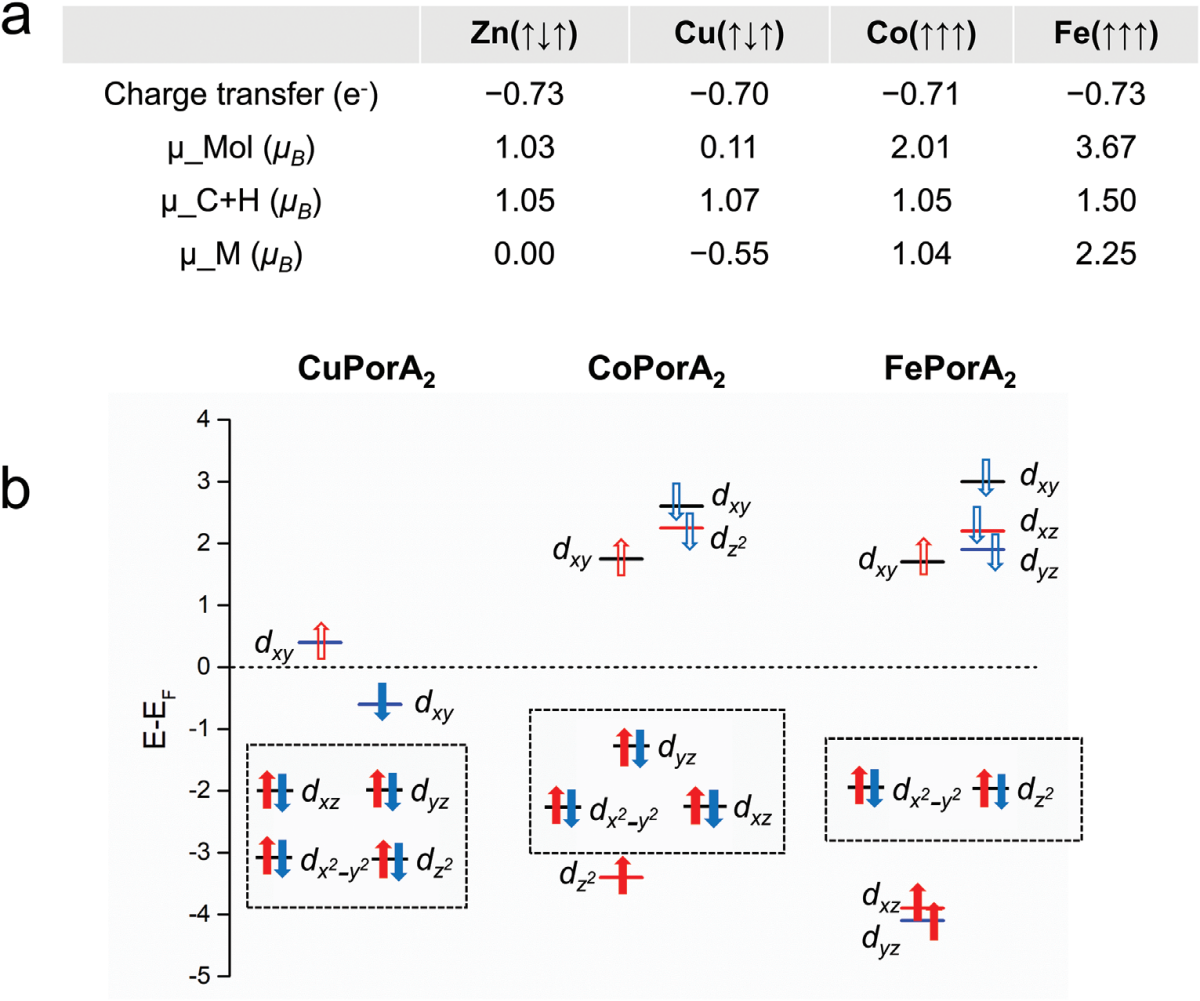
Magnetic properties of **CuPorA_2_**, **CoPorA_2_**, and **FePorA_2_** on Au(111). a) Summarized DFT results of the most stable states of **CuPorA_2_**, **CoPorA_2_**, and **FePorA_2_** on Au(111). The arrows in the brackets indicate the spin orientations of the left and right edges and the central metal. µ_Mol is the magnetic moment of the entire molecule (i.e., ligand and metal center), µ_C+H is the magnetic moment of the ligand part, and µ_M is the magnetic moment of the transition metal center. The signs of the magnetic moments indicate the spin directions. b) Schemes describing the *3d* level occupation of Cu^II^, Co^II^, and Fe^II^ in **CuPorA_2_**, **CoPorA_2_**, and **FePorA_2_**, respectively, based on the projected density of states (PDOS) results (Figure S2.7, Supporting Information). The empty arrows stand for the unoccupied orbitals.

Therefore, the conductance step observed for **CuPorA_2_** and the resulting *dI*/*dV* spectrum can be attributed to spin excitations from a ferromagnetic to an antiferromagnetic coupling of the edge spins, as indicated in Figure 2d. The STS maps of **CuPorA_2_** that are acquired slightly above the excitation threshold (at ±25 mV) show that the spin density is strongly located over the edges, in agreement with the computed spin density distribution of the edge electrons. As shown in Figure 3b, the magnetic coupling between the Cu ion and the edges is weak (2 meV). Therefore, the inelastic steps and the Kondo resonance observed at the STS curve over the edges are strictly localized at the edges and they are not observed over the Cu ion. This localization can also be visualized in the STS maps (Figure 2g). These spectral features, which are similar to those reported for the closed‐shell **ZnPorA_2_**, can be rationalized by the contribution of the d_xy_ orbital to the local magnetic moment of **CuPorA_2_** (**Figure** **4**b). This orbital lies parallel to the surface and interacts poorly with the STM tip. Therefore, its detection in the STS spectra taken over the Cu atom is unlikely. This hypothesis is also consistent with the depression at the Cu position in the topographic STM image (Figure 1c). At the same time, the direct coupling between the Cu d_xy_ orbital and the surface is also expected to be weak and the expected Kondo temperature associated with the Kondo effect would be much lower than the temperature of our experiments, making it undetectable in these conditions. Finally, the other possible difference between the Cu and the ZnPors would be the spin coupling between Cu and the edges, which is absent for Zn. However, this coupling is weak (≈2 meV according to our calculations, see Figure 3b) and difficult to be detected in the conditions of our experiments.

In the case of **FePorA_2_**, the conductance steps at biases of ±21.0 mV are attributed to the transition from ferromagnetic to the antiferromagnetic coupling of the edge spins. Due to conservation of the total spin, spin excitations—induced by inelastic electron tunneling—involve only spin states whose total spin quantum number differs by zero or one.^[^ ^67^ ^]^ The **FePorA_2_** molecule has a spin of 2 (see results in Figure 4 and the Supporting Information) and the low‐energy excitation seen at ±5.6 mV involves an excited state of *S*_z_ = 1. Due to the large interaction between edge and Fe spins, this excitation is likely due to the local magnetic anisotropy of the Fe^II^ ion. To gain more insight on the conductance step at smaller energies for **FePorA_2_**, we have carried out several calculations including spin‐orbit coupling, which allow a prediction of the MAE. We found a theoretical MAE of ≈2 meV, with the magnetic moment lying within the plane of the molecule, which could explain the steps at ±5.6 mV. Consequently, the spectra of **FePorA_2_** can be interpreted as a superposition of the spin excitation between the edge spins (*J*_*π*–*π*_) and the MAE of the Fe electrons (*D*), as illustrated in Figure 2f. The quantitative discrepancies between the experimental (i.e., 21 meV, Figure 2c) and theoretical exchange coupling energy (i.e., 35–37 meV, Figure 3b) arise from the static character of the theoretical calculations, while the experimental results are obtained by measurements of electronic transport currents. This is a known limitation of the PBE‐based DFT calculations, which struggle to quantitatively describe such magnetic systems, as discussed for **ZnPorA_2_**.^[^ ^55^ ^]^

For **CoPorA_2_**, we do not find any conductance step, which seems contradictory to theoretical predictions. To address this issue, we performed further DFT analysis on the three metalated Pors adsorbed on Au(111), as displayed in Figure 4. In all three cases, and in good agreement with the previously reported **ZnPorA_2_**,^[^ ^55^ ^]^ a charge transfer of ≈0.7 electrons from the Por to the surface is predicted. Notably, the amount of charge transfer is largely independent of the nature of the metal ion and does not affect the magnetic features of the ground states. The magnetic moments of the organic part of the Por (*μ_*C+H), which represent the contribution of the *π‐*magnetism, are all ≈1 *μ_B_* for **ZnPorA_2_**, **CuPorA_2_**, and **CoPorA_2_** and somewhat higher for **FePorA_2_** at 1.5 *μ_B_*. Regarding the metal ions (*μ_*M), the Cu^II^ and Co^II^ ions carry −0.55 and 1 *μ_B_*, respectively, whereas Fe^II^ outstands with a magnetization of 2.25 *μ_B_*.

To clarify the magnetic contributions of the metals, we have plotted their PDOS on the 3*d* orbitals (Figure S2.7, Supporting Information) and sketched the simplified 3*d* level occupations of Cu^II^, Co^II^, and Fe^II^ in Figure 4b, based on the PDOS results. The magnetic moment of the Cu^II^ ion derives from the singly occupied *d*_xy_ orbital, while in Co^II^ it stems from the singly occupied *d*_z2_ orbital. This latter *d*_z2_‐derived orbital suffers more broadening than the *d*_xy_‐derived states as a consequence of its larger hybridization with the substrate. Therefore, a stronger Kondo screening between the CoPor and the substrate is expected due to the dominant spin polarization from the *d*_z2_ orbital. Indeed, Co‐Pors on Au(111) usually exhibit intense zero‐bias resonances due to Kondo screening.^[^ ^68^, ^69^ ^]^

The spatial distribution of the Co‐derived Kondo screening can be extended into the Por ligand.^[^ ^68^, ^70^ ^]^ Since the Kondo temperatures of the spectra over the edge and Co ion in **CoPorA_2_** are similar, we attribute both the Kondo resonance observed on the edge and on the central metal ion to the Co‐derived Kondo screening (Figure 2b). Thus, we speculate that the “expected” spin excitation between the edge spins and the resulting conductance steps is suppressed due to the intense Kondo resonance. The experimental Kondo map of **CoPorA_2_**, acquired by constant‐height STM imaging at –10 mV, shows contrast over both the center and edges and is compatible with the proposed scenario (Figure 2h).

## 4 Conclusion

We have reported the in‐solution and on‐surface fabrication of three open‐shell, *π*‐extended Pors with magnetically active metal ions in their inner cavity (i.e., Cu^II^, Co^II^, and Fe^II^, respectively). After the unambiguous structural confirmation of the three surface‐supported nanostructures, the magnetic properties of these metalloPors were carefully addressed by high‐resolution scanning probe microscopy, complemented by DFT calculations at the single‐molecule level.

Our detailed study, which focuses on the magnetic interplay between the *π*‐ and *d‐* electrons, reveals that the *π*‐spin of the edges of the metalloPors are ferromagnetically coupled in their ground states, as reflected by conductance steps in the STS of **CuPorA_2_** and **FePorA_2_**. Meanwhile, the low‐bias STS spectra of **CoPorA_2_** are dominated by a Kondo resonance resulting from the singly occupied Co^II^ $d_{z^{2}}$ orbital. Among the three Pors, **FePorA_2_** exhibits the highest magnetization of 3.67 *μ_B_* (*S* ≈ 2), with 2.25 *μ_B_* for the Fe ion, and a directly observed MAE of 5.6 meV. Moreover, **FePorA_2_** shows a relatively strong exchange coupling of 16 meV between the ligand *π‐*electrons and the Fe *d*‐states.

In summary, the herein presented work provides a facile method for the fabrication of open‐shell organic systems that contain chelated magnetically active transition metal ions. As demonstrated in this detailed study, the *π*‐electrons of the Por macrocycle can have a strong magnetic interplay with the *d‐*electrons of the central metal ion in our metalloPors. Similar interactions have been explored in the literature, sometimes giving rise to remarkable properties such as superconductivity,^[^ ^71^ ^]^ semiconductor‐to‐metal transition,^[^ ^72^ ^]^ and high electrocatalytic activity.^[^ ^13^, ^73^, ^74^ ^]^ Therefore, our remarkably straightforward method may pave the way toward the realization of fascinating novel materials with intriguing magnetic properties.

In this context and taking advantage of a previously reported methodology,^[^ ^56^ ^]^ the next logical step would be the lateral fusion of the here reported magnetically active MPorA_2_. The resulting metalloPor NTs would contain multiple magnetically active metal ions in a short distance due to the close vicinity of the inner cavities of the fused Pors. This could allow the magnetic interaction of the unpaired *d*‐electrons of the metal ions, leading to new and exciting features. The synthesis and characterization of CuPor NTs, CoPor NTs, and FePor NTs is currently being carried out in our laboratories and will be reported in due course.

## 5 Experimental Section

### STM/STS and nc‐AFM characterization

A commercial low‐temperature STM/AFM system (Scienta Omicron) was used for the preparation and in situ characterization of samples. Experiments were performed under UHV conditions (base pressure ≈ 2 × 10^‐^^10^ mbar). Au(111) single crystal substrates were cleaned by cycles of standard argon sputtering and annealing. Deposition of the molecular precursors was carried out by thermal evaporation from a sixfold organic evaporator (Mantis GmbH). STM images were recorded at liquid helium temperature of 4.5 K in constant‐current mode unless specified otherwise, and the d*I*/d*V* spectra were recorded using the lock‐in technique (U_RMS_ = 20 mV for wide‐range spectra and U_RMS_ = 0.8–1 mV for low‐bias spectra). The low‐bias spectra shown were averages of 8–12 consecutive single spectra. nc‐AFM images were recorded with a CO‐functionalized tip^[^ ^75^ ^]^ attached to a quartz tuning fork sensor^[^ ^76^ ^]^ (resonance frequency 23.5 kHz, peak‐to‐peak oscillation amplitude below 100 pm). The data were processed and analyzed with Wavemetrics Igor Pro or WSxM software. Fitting of spin excitation spectra was performed with the program provided in ref. [64]. Kondo resonances were fitted using Wavemetrics Igor Pro software.

### Theoretical Methods

DFT calculations were performed using the VASP code^[^ ^77^ ^]^ and the PBE exchange and correlation functional.^[^ ^78^ ^]^ For Co and Fe, the GGA+U method^[^ ^79^ ^]^ was used with U–J = 4 eV for Co^[^ ^70^ ^]^ and U–J = 3 eV for Fe.^[^ ^80^ ^]^ Additional calculations for the gas‐phase molecules were done with the B3LYP hybrid functional.^[^ ^81^ ^]^ Core electrons were treated within the projector augmented method^[^ ^82^, ^83^ ^]^ and wavefunctions were expanded using a plane‐wave basis set with an energy cut‐off of 400 eV. The (111) surface of Au was simulated using a 4‐layer slab and a vacuum gap of 18 Å. The two bottom layers of Au were kept fixed and all the other atoms were relaxed until forces were smaller than 0.02 eV Å^−1^ (0.001 eV Å^−1^ for the gas‐phase calculations). A (03 × 02 × 01) k‐grid mesh was used for the sampling of the Brillouin zone. van der Waals interactions were included using the Tkatchenko–Scheffler scheme.^[^ ^84^ ^]^ Theoretical STM images were simulated within the Tersoff–Hamann method^[^ ^85^ ^]^ as described by Bocquet et al.^[^ ^86^ ^]^ and implemented in the STMpw program.^[^ ^87^ ^]^

## Conflict of Interest

The authors declare no conflict of interest.

## Author Contributions

Q.S., L.M.M., and R.R. contributed equally to this work. Q.S. performed the on‐surface synthesis and STM/STS measurements under the supervision of P.R. and R.F.. L.M.M. synthesized the molecular precursors, under the supervision of T.T. and G.B.. R.R. and N.L. carried out the DFT calculations. Q.S., L.M.M., G.B., and R.R. wrote the paper with contributions from all co‐authors. All authors discussed the results and commented on the manuscript at all stages.

## Supporting information

Supporting Information

## Data Availability

The data that support the findings of this study are available from the corresponding author upon reasonable request.
