# Supplementary material for: Magnetic Interplay between π‐Electrons of Open‐Shell Porphyrins and d‐Electrons of Their Central Transition Metal Ions
Source: Adv Sci (Weinh). 2022 Mar 18;9(19):2105906. doi: 10.1002/advs.202105906 (PMC9259720; doi:10.1002/advs.202105906)
Supplement: Supplementary file 1 — Supporting Information [file ADVS-9-2105906-s001.pdf]

ADVANCED SCIENCE
Open Access

# Supporting Information

for *Adv. Sci.*, DOI 10.1002/advs.202105906

Magnetic Interplay between $\pi$-Electrons of Open-Shell Porphyrins and *d*-Electrons of Their Central Transition Metal Ions

*Qiang Sun, Luis M. Mateo, Roberto Robles, Pascal Ruffieux, Giovanni Bottari*, Tomás Torres*, Roman Fasel* and Nicolás Lorente**

*Supporting Information for*

# "Magnetic interplay between *π*-electrons of open-shell porphyrins and *d*-electrons of their central transition metal ions"

Qiang Sun,[⊥,ǂ,¶] Luis M. Mateo,[†,‡,¶] Roberto Robles,[ǁ,¶] Pascal Ruffieux,[⊥] Giovanni Bottari,*[,†,‡,§] Tomás Torres*[,†,‡,§], Roman Fasel*[,⊥,+] and Nicolas Lorente,[ǁ,º,*]

⊥ nanotech@surfaces Laboratory, Empa-Swiss Federal Laboratories for Materials Science and Technology, 8600 Dübendorf, Switzerland.

† Departamento de Química Orgánica, Universidad Autónoma de Madrid, 28049 Madrid, Spain

‡ IMDEA-Nanociencia, Campus de Cantoblanco, 28049 Madrid, Spain.

ǁ Centro de Física de Materiales CFM/MPC (CSIC-UPV/EHU), Paseo de Manuel de Lardizabal 5, 20018 Donostia-San Sebastián.

º Donostia International Physics Center (DIPC), 20018 Donostia-San Sebastián, Spain.

§ Institute for Advanced Research in Chemical Sciences (IAdChem), Universidad Autónoma de Madrid, 28049 Madrid, Spain.

ǂ Materials Genome Institute, Shanghai University, 200444 Shanghai, China.

\+ Department of Chemistry, Biochemistry and Pharmaceutical Sciences, University of Bern, 3012 Bern, Switzerland.

¶ These authors contributed equally to the paper

E-mail: roman.fasel@empa.ch (R.F.); tomas.torres@uam.es (T.T); giovanni.bottari@uam.es (G.B.); nicolas.lorente@csic.es (N.L.)

## Table of Content

1. General: Materials and Methods ............ 3
2. Additional on-surface characterization and DFT calculations ............ 4
3. Solution-based synthesis and characterization ............ 9
Synthesis and characterization of 5,15-bis(2,6-dimethylphenyl)porphyrinato Cu(II) **CuPor(dmp)$_2$** .. 9
Synthesis and characterization of 5,15-bis(2,6-dimethylphenyl)porphyrinato Co(II) **CoPor(dmp)$_2$** 13
Synthesis and characterization of 5,15-bis(2,6-dimethylphenyl)porphyrinato Fe(II) **FePor(dmp)$_2$**.. 16
4. References ............ 19

Abbreviations: APCI = atmospheric pressure chemical ionization; ATR = attenuated total reflectance; DCM = dichloromethane; DCTB = *trans*-2-[3-(4-*tert*-butylphenyl)-2-methyl-2-propenylidene]malononitrile; DDQ = 2,3-dichloro-5,6-dicyano-1,4-benzoquinone; DFT = density functional theory; FT-IR = Fourier-transform infrared spectroscopy; MALDI-TOF = matrix-assisted laser desorption/ionization-time of flight; MS = mass spectrometry; nc-AFM = noncontact atomic force microscopy; NMR = nuclear magnetic resonance; PEG = polyethylene glycol; Por = porphyrin; RF = retention factor; RMS = high resolution mass spectrometry; STM = scanning tunneling microscopy; STS = scanning tunneling spectroscopy; THF= tetrahydrofurane.

**1. General: Materials and Methods**

Chemicals and solvents were purchased from commercial suppliers (Aldrich, Fluka, Strem, Acros and Fischer) and used without further purification. All dry solvents were freshly distilled under argon over an appropriate drying agent before use. Column chromatography was carried out on silica gel Merck-60 (230-400 mesh, 60 Å). Analytical TLC was performed on aluminium sheets precoated with silica gel 60 F-254 from Merck. Preparative TLC plates (20 × 20 cm plates) were purchased from Merck (silica gel-60, 0.5 mm). $^{1}H$ and $^{13}C$ NMR spectra were recorded with a Bruker Advance 300 MHz instrument, a Bruker DPX 400 MHz instrument or a Bruker DRX 500 MHz instrument. Chemical shifts values (δ) are referred to tetramethylsilane.

UV/vis experiments were carried out by using quartz cells with a 1 cm optical path length in a Varian Cary 50 UV spectrophotometer. IR spectra were recorded on a Bruker ALPHA Platinum-ATR system.

MALDI-TOF MS spectra were obtained in a Bruker ULTRAFLEX III (MALDI-TOF/TOF) spectrometer. GC-MS spectra were obtained from a Waters GCT Agilent Technologies 6890N spectrometer. APCI-MS spectra were obtained from a Bruker MAXIS II spectrometer.

## 2. Additional on-surface characterization and DFT calculations

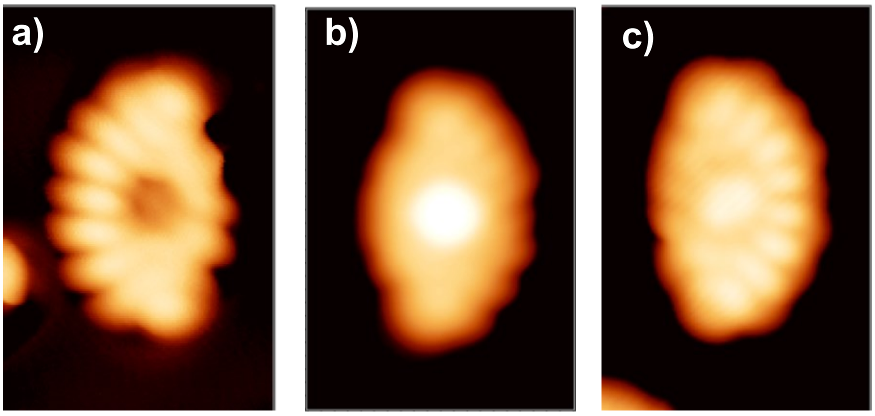


**Figure S2.1.** STM images of a) monoradical **CuPorA2**, a) monoradical **CoPorA2** and a) monoradical **FePorA2** species. Scanning parameters: monoradical **CuPorA2** $V_s$ = –0.05 V, $I_t$ = 100 pA; monoradical **CoPorA2** $V_s$ = –0.06 V, $I_t$ = 100 pA; monoradical **FePorA2** $V_s$ = –0.05 V, $I_t$ = 200 pA.

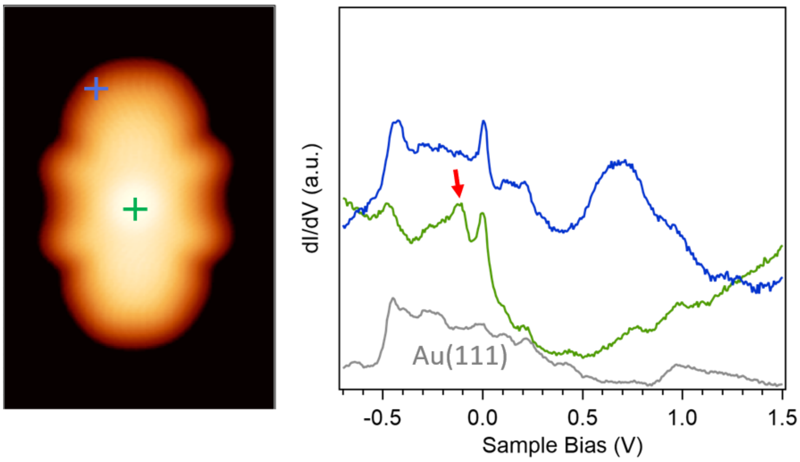


**Figure S2.2.** STM image and wide bias range STS spectra of **CoPorA2** on Au(111). There is a strong and broad resonance peaking at −0.1 V localized over the Co atom, which is indicated by a red arrow. The reference spectrum taken on the bare surface is colored in gray.

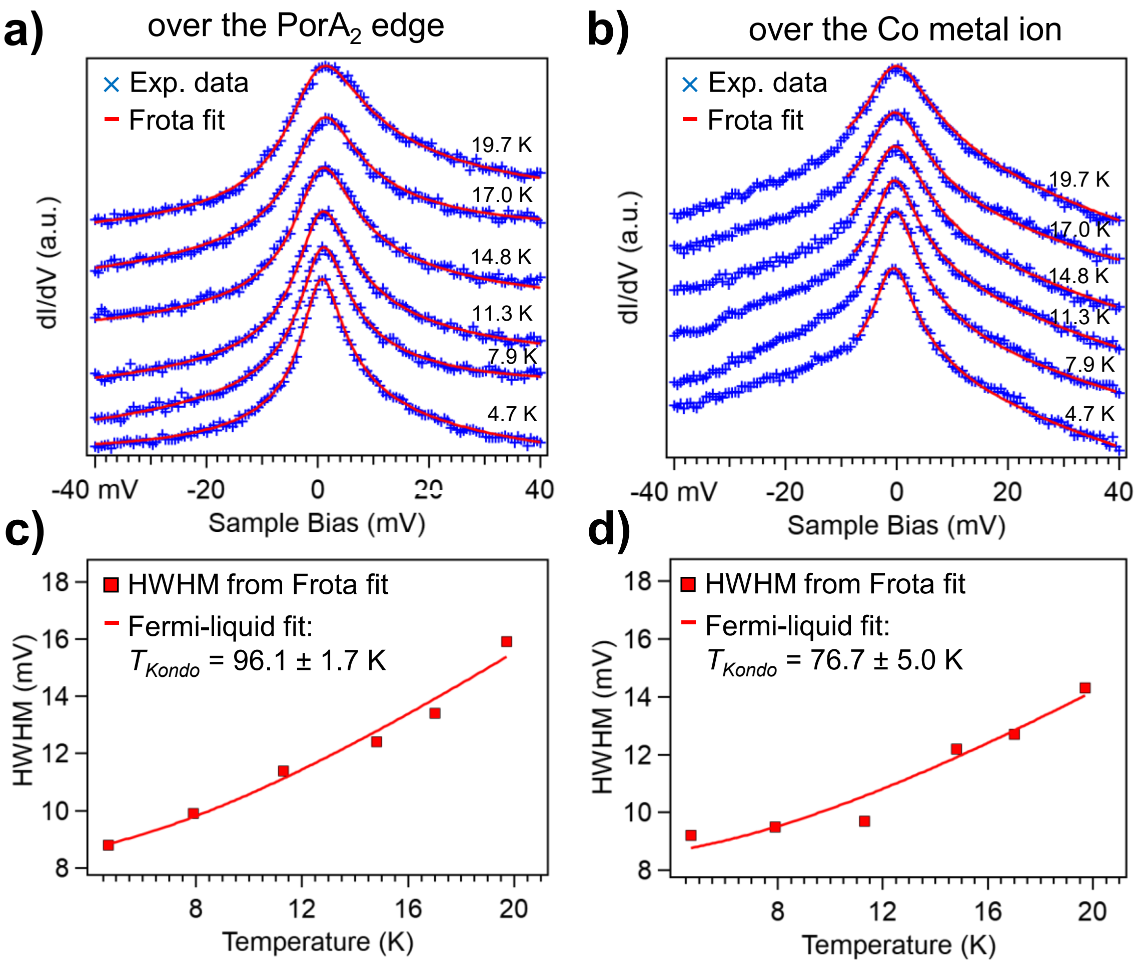


**Figure S2.3.** Temperature evolved STS spectra over a) the Por edge and b) the Co metal ion of **CoPorA2** with the experimental data fit by the Frota function (red lines). Extracted half-widths at half-maximum (HWHM) $\Gamma$ of the Kondo resonance as a function of temperature over c) the Por edge and d) the Co metal ion of **CoPorA2**. The HWHM are fit using the Fermi-liquid model $\Gamma = \frac{1}{2}\sqrt{(\alpha k_B T)^2 + (2k_B T_K)^2}$ to determine the Kondo temperature $T_k$.

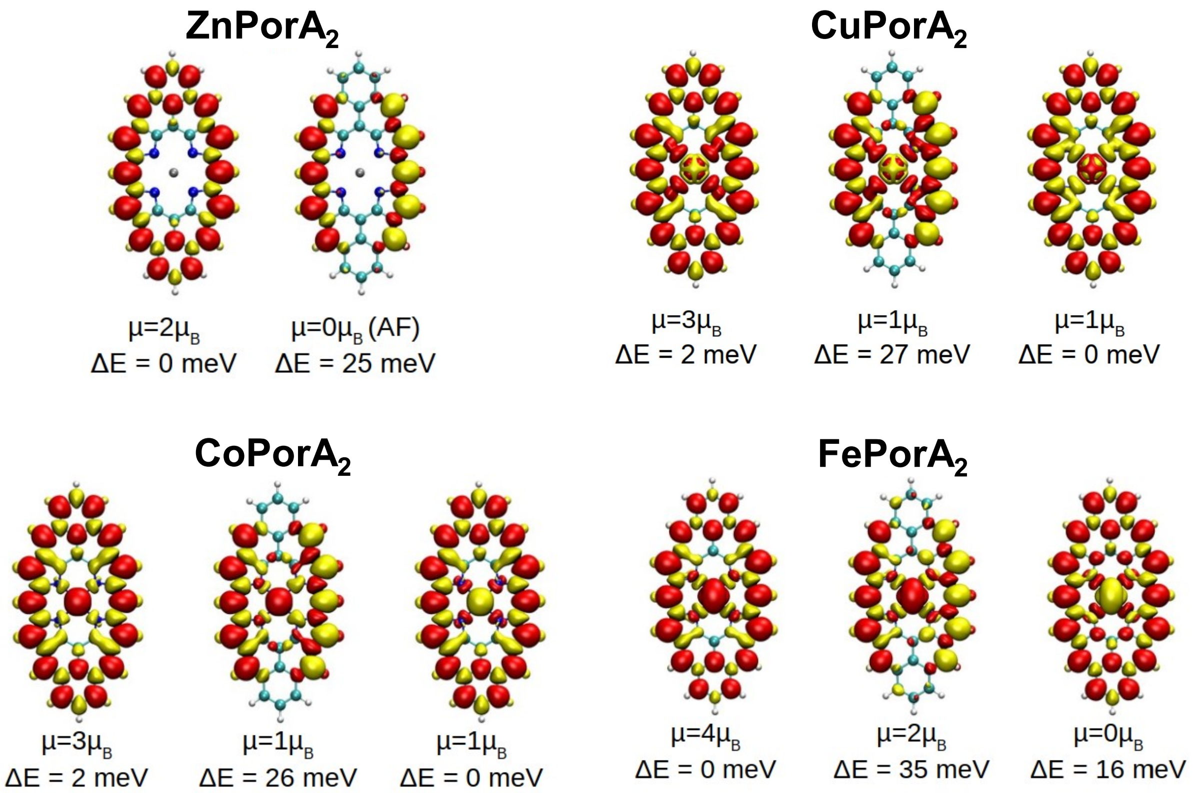


**Figure S2.4.** DFT calculated spin density maps for different magnetic states of **ZnPorA$_2$**, **CuPorA$_2$**, **CoPorA$_2$** and **FePorA$_2$** using the B3LYP hybrid functional. The total magnetic moments and the relative energies of different states are displayed below the corresponding maps. Red and yellow densities denote spin-up and spin-down contributions, respectively.

It can be clearly seen that, based on the spin directions, the spin densities of the metalloPors complexed with magnetically active transition metals (*i.e.*, **CuPorA$_2$**, **CoPorA$_2$** and **FePorA$_2$**) can be divided into three regions, *i.e.* the left edge, the central metal ion and the right edge. The left and right edge regions originate from the unpaired $\pi$ electrons and the central spin densities from the transition metal ion *d*-electrons. Thus, the magnetic coupling among the three regions can result in three different states for the three metalloPors.

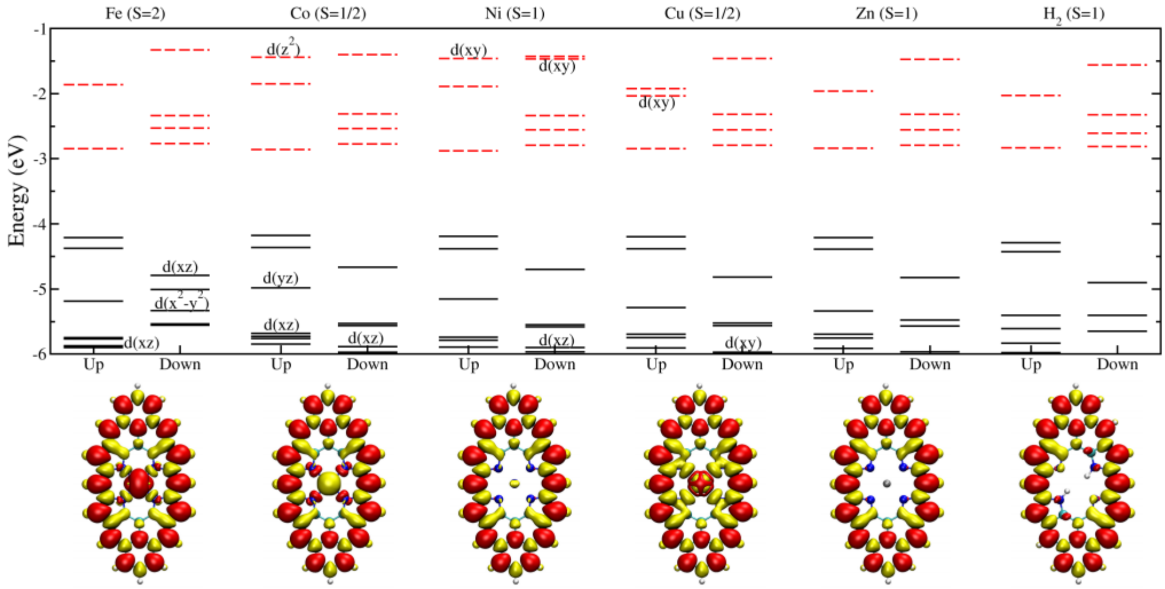


**Figure S2.5.** Upper panel: Spin-polarized energy levels of gas-phase **MPorA$_2$** molecules, with M = Fe, Co, Ni, Cu, Zn and $H_2$, and calculated using the B3LYP functional. Occupied (empty) states are represented with full (dashed) lines. Levels with a significant *d* occupation are labeled. Lower panel: spin density maps of the ground state shown in the upper panel. Red (yellow) densities denote spin-up (spin-down) contributions.

The behavior of the edges is quite similar through the series, with the left and right edges ferromagnetically coupled. For the Fe, Co and Cu Pors the central atom is magnetic. The Fe metal ion couples ferromagnetically to the edges, while the Co and Cu metal ions couple antiferromagnetically. The Fe metal ion has two unpaired electrons ($d_{yz}$ and $d_{z^2}$). The Co and Cu metal ions have one unpaired electron residing on a $d_{z^2}$ orbital for **CoPorA$_2$** and on a $d_{xy}$ orbital for **CuPorA$_2$**.

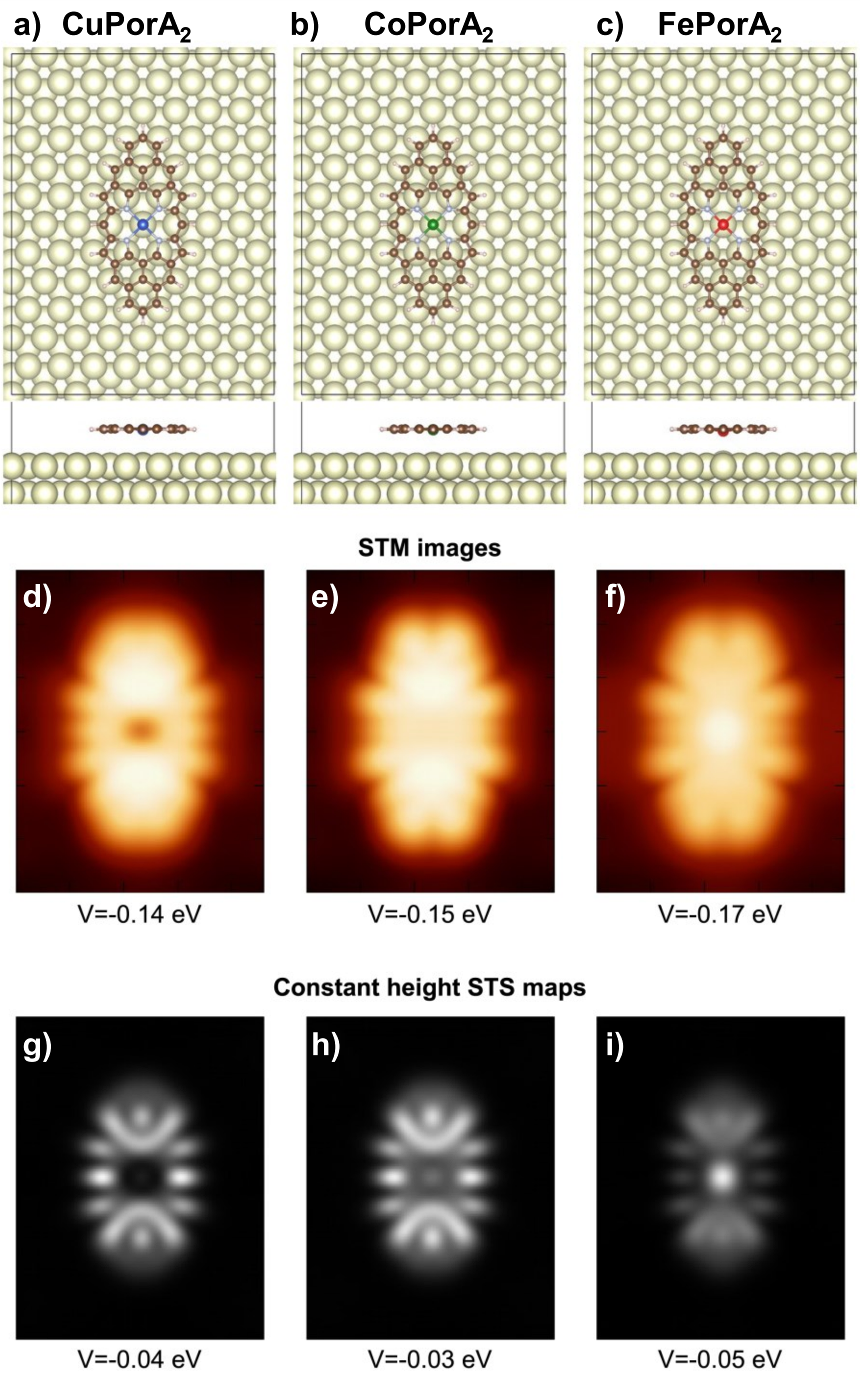


**Figure S2.6.** Upper panel: geometries of the surface-adsorbed Pors a) **CuPorA₂**, b) **CoPorA₂** and c) **FePorA₂** optimized using DFT. Middle panel: simulated topographic STM images of d) **CuPorA₂**, e) **CoPorA₂** and f) **FePorA₂** calculated at small negative biases (occupied states). Lower panel: simulated constant height STS maps of g) **CuPorA₂**, h) **CoPorA₂** and i) **FePorA₂**. For the three metalloPors, the molecules remain planar after deposition, with the transition metal atom lying in the plane of the molecule. Therefore, the lower contrast at the position of the Cu atom (shown in the STM images and STS maps) has an electronic origin. The reason is that for Cu the *d* state around the Fermi energy ($d_{xy}$) is parallel to the surface and has a small extension into the vacuum and, consequently, a small coupling with the tip states.

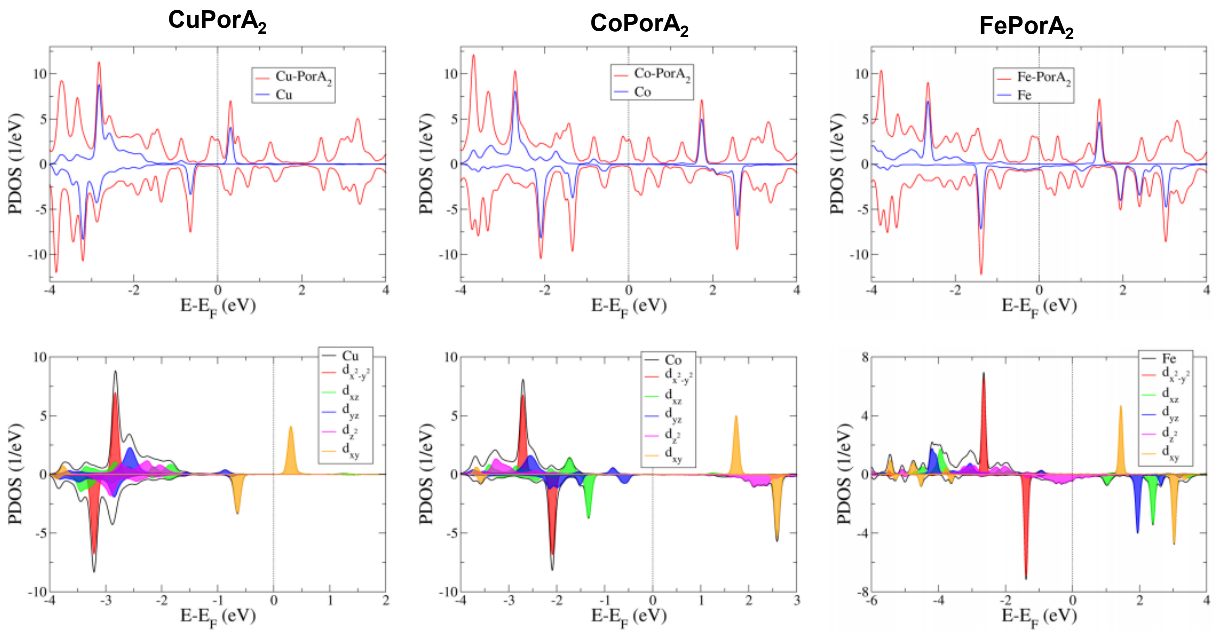


**Figure S2.7.** Upper panel: projected densities of states (PDOS) for **$CuPorA_2$** (left), **$CoPorA_2$** (center) and **$FePorA_2$** (right) supported on Au(111). Lower panel: PDOS for the transition metal atoms of the molecules above.

The hybridization of the molecule with the substrate can be seen in the width of the levels with a perpendicular (z) component, in particular $d_{z^2}$, while the levels which lie parallel to the surface are more atomic-like.

## 3. Solution-based synthesis and characterization

### Synthesis and characterization of 5,15-bis(2,6-dimethylphenyl)porphyrinato Cu(II) CuPor(dmp)$_2$

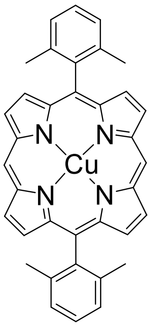


**$H_2$Por(dmp)$_2$** (40 mg, 0.0771 mmol) and $Cu(OAc)_2$ (280 mg, 1.54 mmol, 20 eq.) were loaded in a flame-dried 50 mL round-bottomed flask and dissolved in THF (15 mL). The mixture was heated to reflux and stirred overnight under argon. The mixture was then allowed to cool to room temperature and the solvent was removed under reduced pressure. The crude product was

then dissolved in DCM (100 mL), washed with water (2 × 100 mL) and brine (100 mL), dried over anhydrous $MgSO_4$, filtered and dried under reduced pressure. The solvents were evaporated under reduced pressure and the crude product was further purified by size exclusion chromatography (BioBeads, $CHCl_3$), where the intense red fraction was collected. The solvent was reduced to a small amount under reduced pressure and the crude product was passed through a short plug of $SiO_2$ gel ($CHCl_3$). The solvent was removed under reduced pressure and the resulting crude product was suspended in MeOH, sonicated, filtered, washed with MeOH (10 mL), collected and dried under vacuum to yield **CuPor(dmp)$_2$** as a purple solid (38.5 mg, 86%).

**RF** = 0.78 (3:2 DCM/*n*-heptane); **$^1$H NMR** and **$^{13}$C NMR** were unsuccessful due to the paramagnetic nature of **CuPor(dmp)$_2$**; **MALDI-TOF MS** (DCTB matrix): m/z 579.1614-583.1682 $[M+H]^{\bullet+}$; **HRMS** (MALDI-TOF, DCTB matrix + PEGMeNa 350 + PEGMeNa 550): calcd for $C_{36}H_{29}N_4Cu$ $[M+H]^{\bullet+}$: 579.1610; found: 579.1614; **UV/vis** ($CHCl_3$): $\lambda_{max}$ (log ε) = 403 (5.72), 528 (4.24), 563 (3.65); **FT-IR (ATR)** ν ($cm^{-1}$) = 2959, 2918, 2362, 1578, 1545, 1519, 1462, 1437, 1384, 1314, 1283, 1212, 1057, 994, 851, 776, 729.

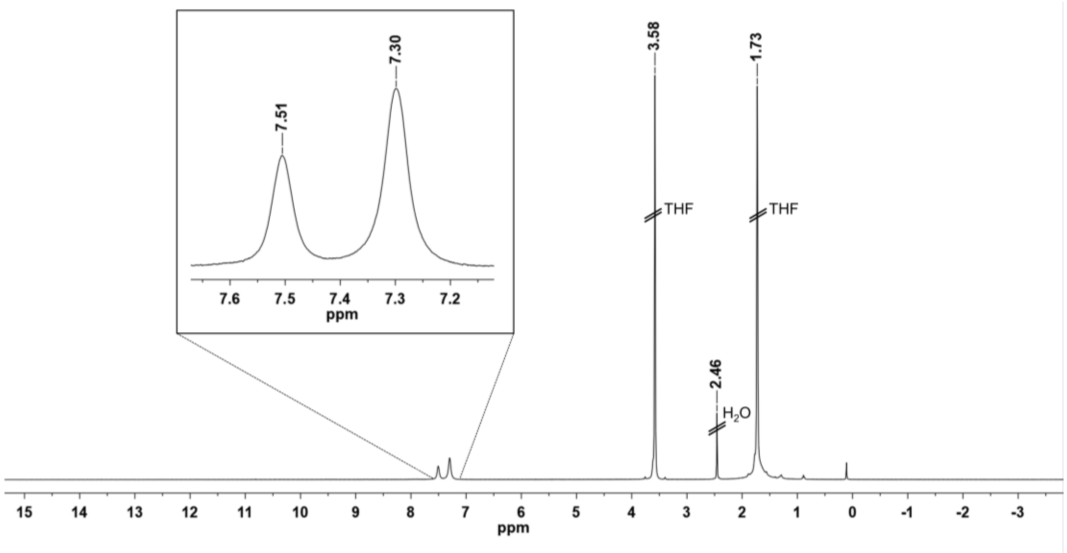


**Figure S3.1.** $^1$H-NMR spectrum of **CuPor(dmp)$_2$** in THF-$d_8$.

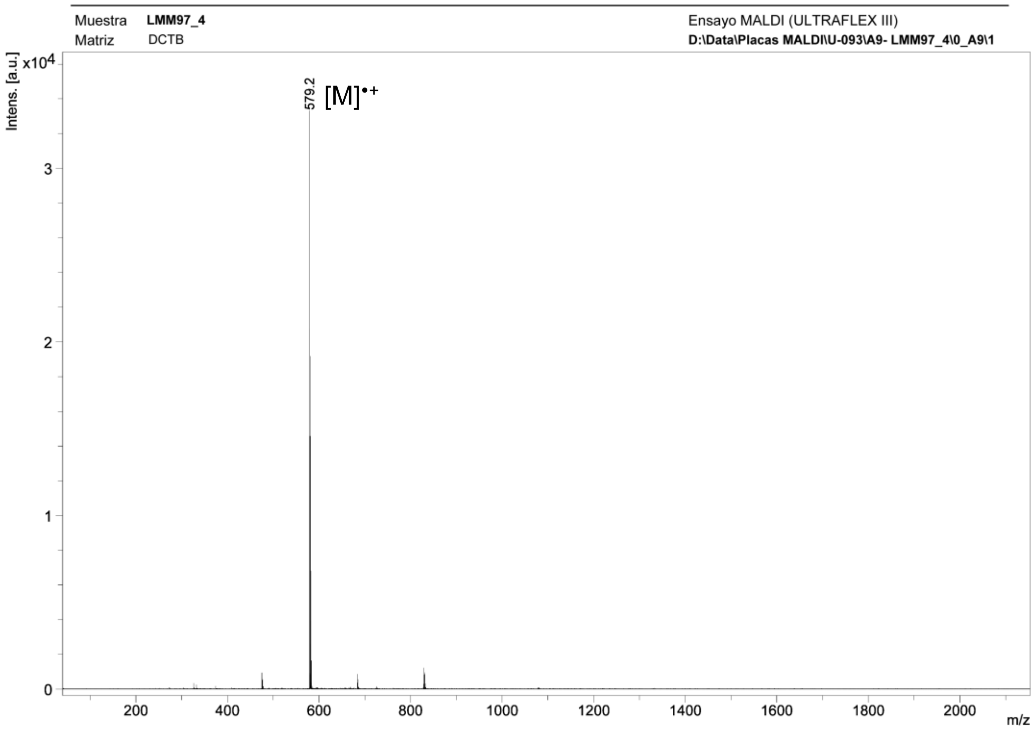


**Figure S3.2.** MALDI-TOF mass spectrum of **CuPor(dmp)$_2$**.

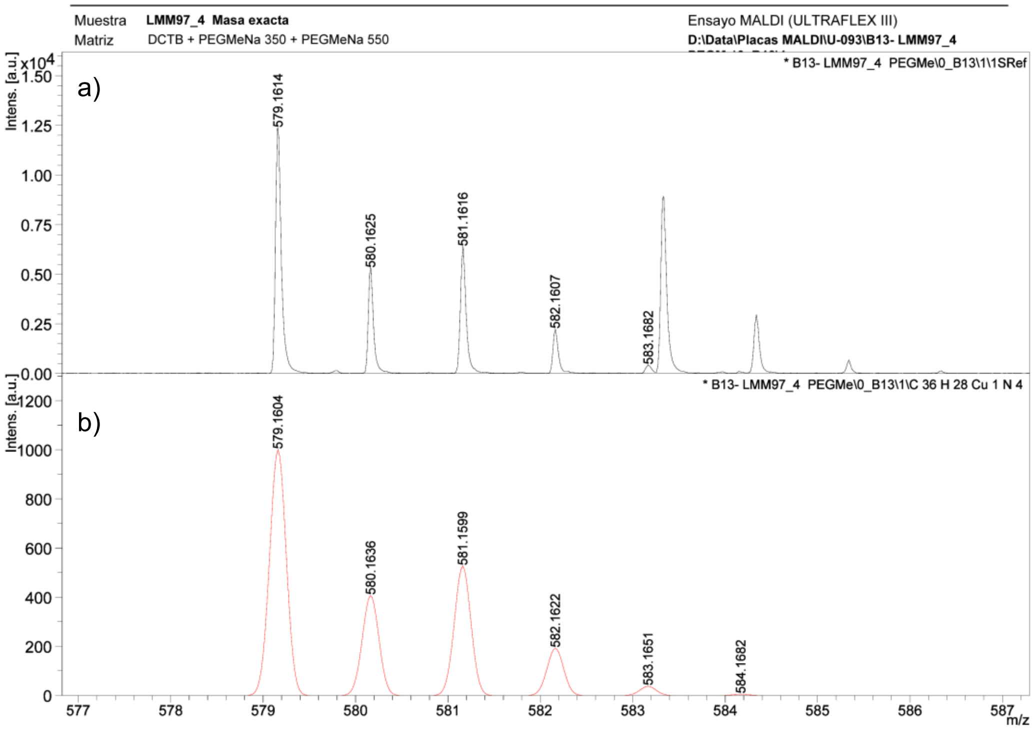


**Figure S3.3.** MALDI-TOF HR mass spectrum of **CuPor(dmp)$_2$**. a) Isotopic distribution of the MALDI-TOF peaks between 579.1614 and 583.1682 m/z; b) calculated isotopic pattern for **CuPor(dmp)$_2$**.

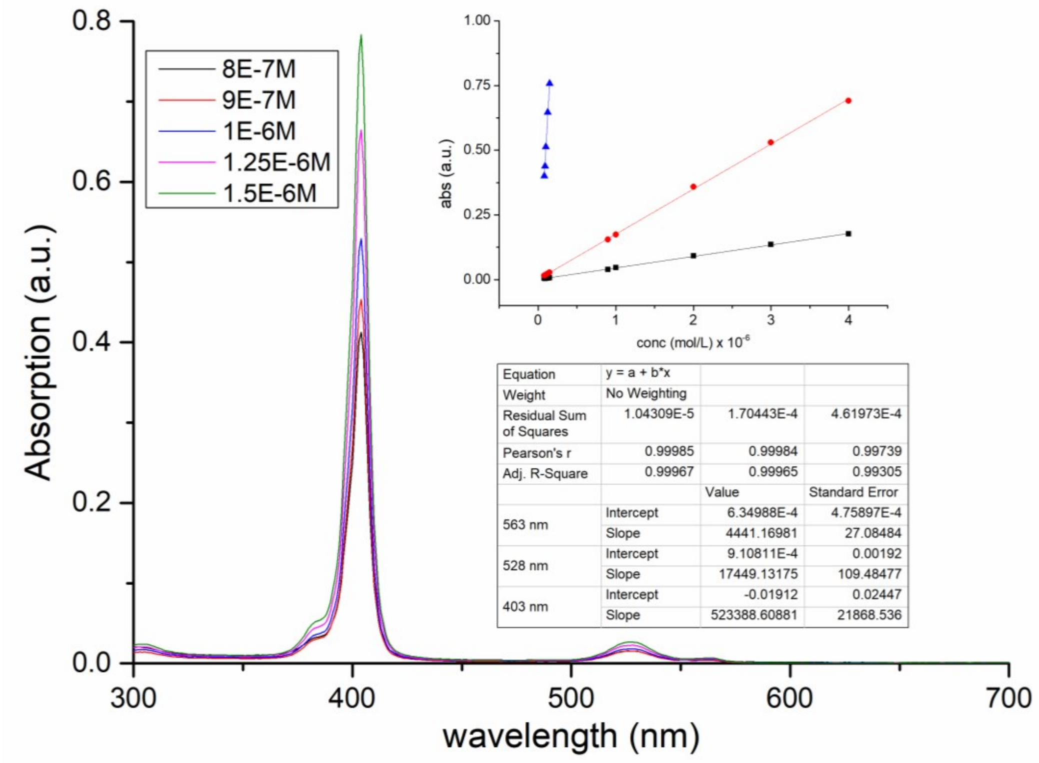


**Figure S3.4.** UV-Vis spectrum of **CuPor(dmp)₂** in $CHCl_3$.

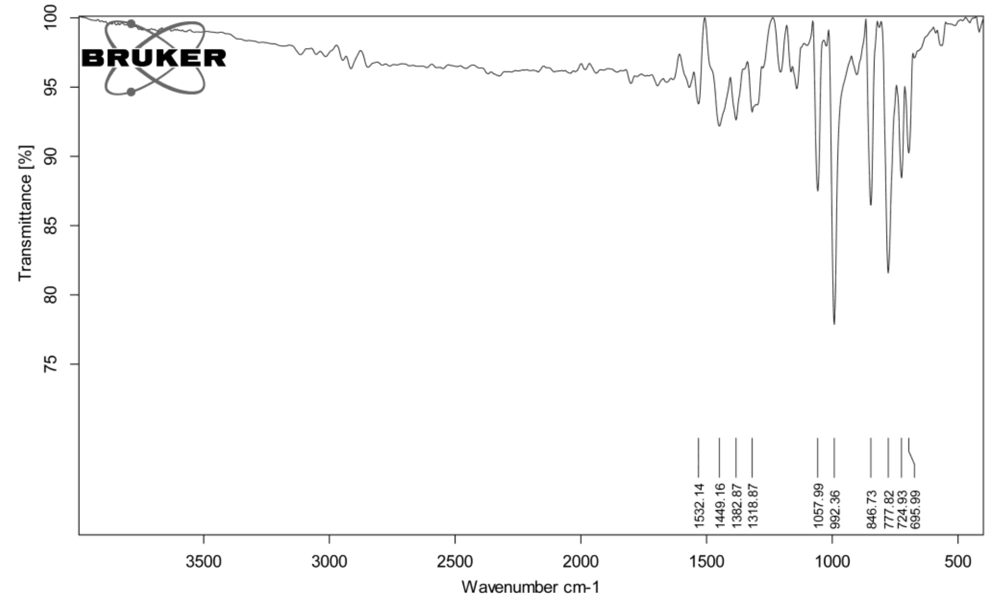


**Figure S3.5.** FT-IR spectrum of **CuPor(dmp)₂**.

**Synthesis and characterization of 5,15-bis(2,6-dimethylphenyl)porphyrinato Co(II) CoPor(dmp)$_2$**

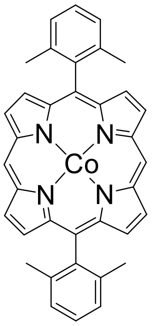


**$H_2$Por(dmp)$_2$** (20 mg, 0.03855 mmol) and $Co(OAc)_2{\cdot}4H_2O$ (47 mg, 0.1927 mmol, 5 eq.) were loaded in a flame-dried 50 mL schlenk tube and subjected to three cycles of vacuum/argon backfilling. Then, DMF (5 mL) was added and the mixture was stirred at 100 °C overnight under argon. The mixture was then allowed to cool to room temperature and the solvent was removed under reduced pressure. The crude product was then dissolved in DCM (100 mL), washed with water (2 × 100 mL) and brine (100 mL), dried over anhydrous $MgSO_4$, filtered and dried under reduced pressure. The solvents were evaporated under reduced pressure and the crude product was further purified by size exclusion chromatography (BioBeads, THF), where the intense red fraction was collected. The solvent was evaporated under reduced pressure, then the crude product was dissolved in $CHCl_3$ and passed through a short plug of $SiO_2$ gel ($CHCl_3$). The solvent was removed under reduced pressure and the resulting crude product was suspended in MeOH, sonicated, filtered, washed with MeOH (10 mL), collected and dried under vacuum to yield **CoPor(dmp)$_2$** as a purple solid (38.5 mg, 86%).

**RF** = 0.74 (2:1 DCM/*n*-heptane); **$^1$H NMR** and **$^{13}$C NMR** were unsuccessful due to the paramagnetic nature of the inner $Co^{II}$ metal ion; **MALDI-TOF MS** (DCTB matrix): 575.1634-577.1698 m/z $[M+H]^{\bullet+}$; **HRMS** (MALDI-TOF, DCTB + PEGMeNa 350 + PEGMeNa 550): calcd for $C_{36}H_{29}N_4Co$ $[M+H]^{\bullet+}$: 575.1646; found: 575.1634; **UV/vis** ($CHCl_3$): $\lambda_{max}$ (log ε) = 400 (5.42), 518 (4.10), 548 (2.77); **FT-IR (ATR)** ν ($cm^{-1}$) = 2953, 2918, 2850, 1541, 1456, 1378, 1314, 1251, 1140, 1060, 991, 847, 777, 724, 696.

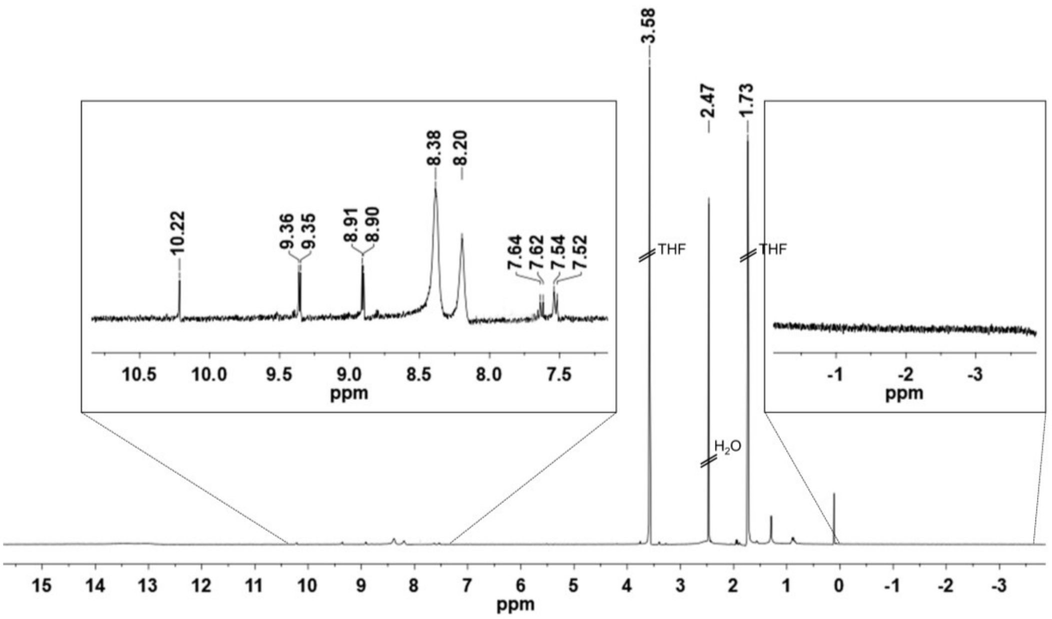


**Figure S3.6.** $^{1}$H-NMR spectrum of **CoPor(dmp)$_2$** in $CDCl_3$. Due to the paramagnetic nature of the inner $Co^{II}$ metal ion, the peak assignment is rather complex and cannot be carried out unambiguously.

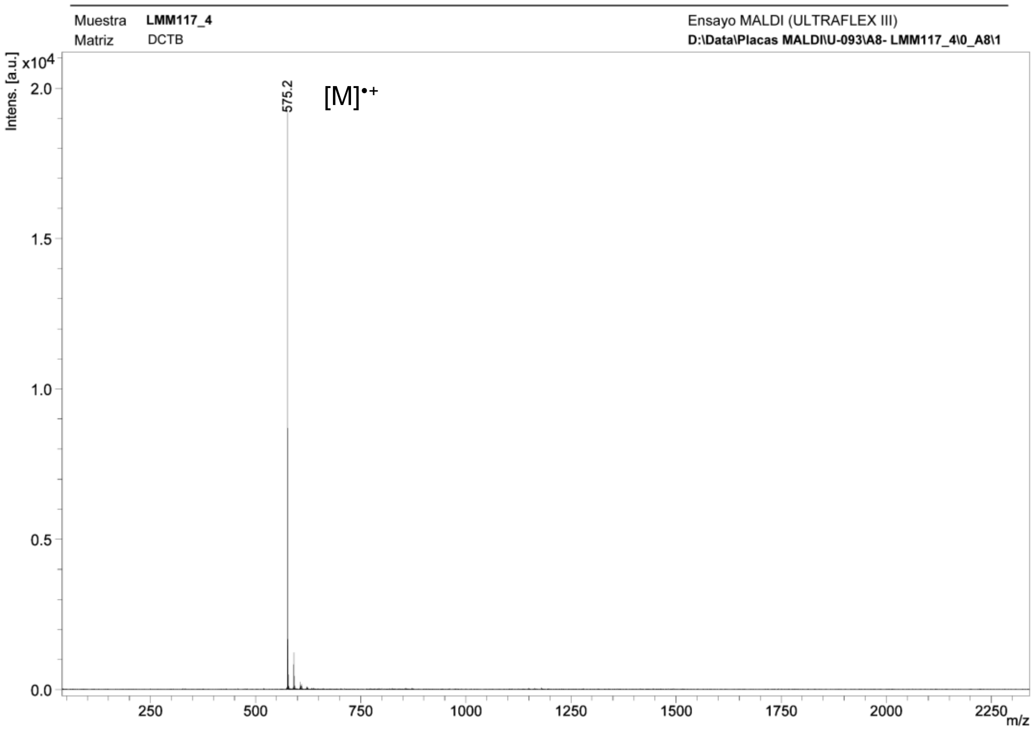


**Figure S3.7.** MALDI-TOF mass spectrum of **CoPor(dmp)$_2$**.

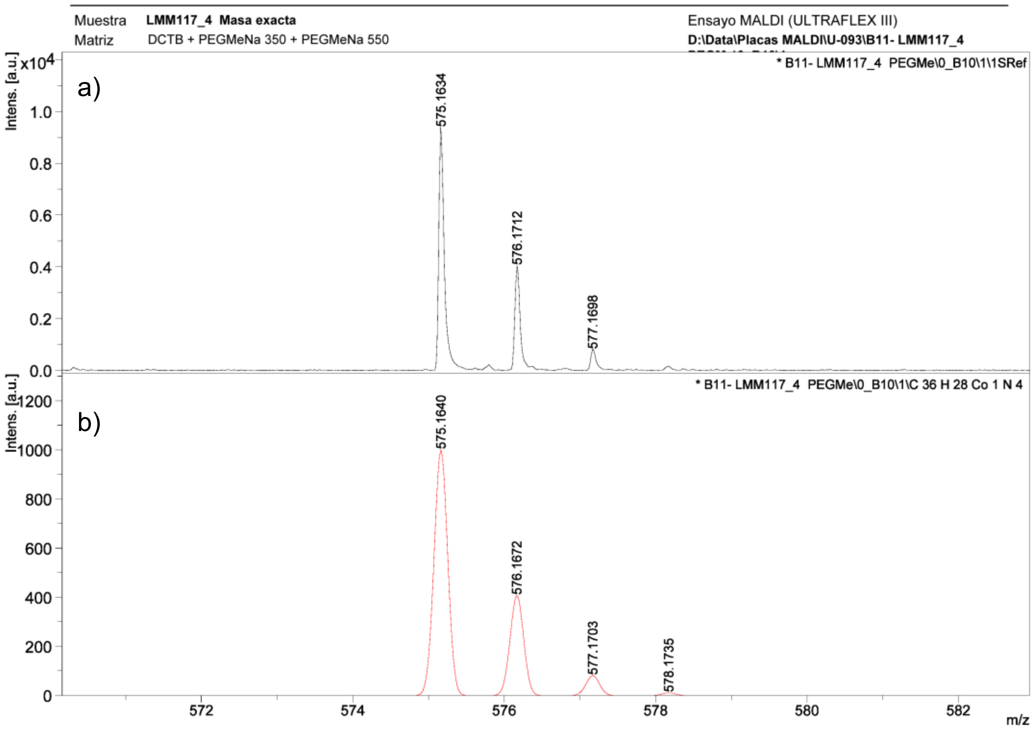


**Figure S3.8.** MALDI-TOF HR mass spectrum of **CoPor(dmp)$_2$**. a) Isotopic distribution of the APCI peaks between 575.1634 and 577.1698 m/z; b) calculated isotopic pattern for **CoPor(dmp)$_2$**.

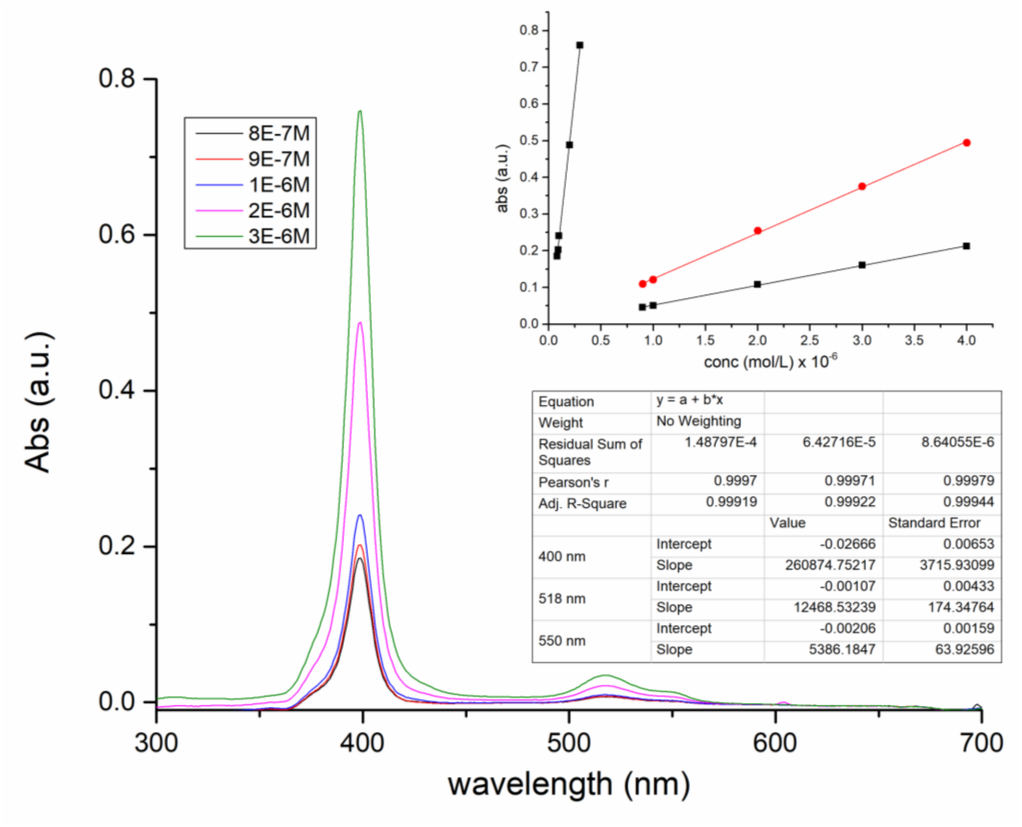


**Figure S3.9.** UV-Vis spectrum of **CoPor(dmp)$_2$** in THF.

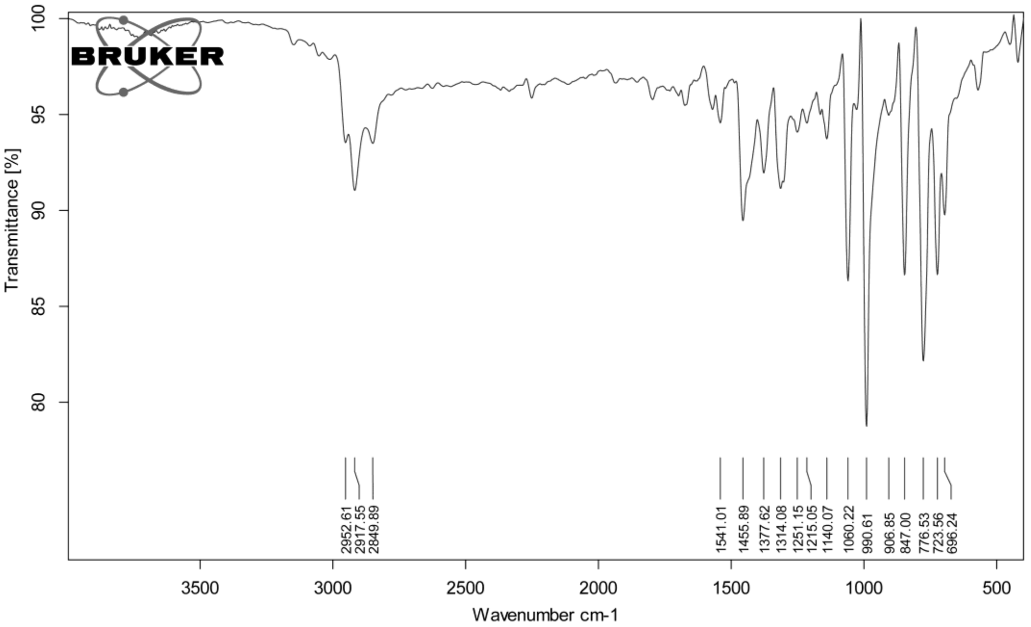


**Figure S3.10.** FT-IR spectrum of **CoPor(dmp)₂**.

**Synthesis and characterization of 5,15-bis(2,6-dimethylphenyl)porphyrinato Fe(II) FePor(dmp)₂**

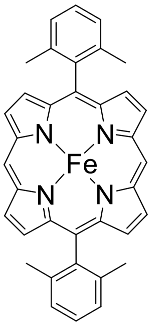

**$H_2$Por(dmp)$_2$** (30 mg, 0.0578 mmol) was loaded in a flame-dried 50 mL Schlenk tube and subjected to three cycles of vacuum/argon backfilling. Then, anhydrous DMF (10 mL) were added under argon and the mixture was heated to 120 °C. $FeCl_2$ (74 mg, 0.578 mmol, 10 eq.) was then added in one batch and the mixture was stirred overnight at this temperature. After cooling to room temperature, the solvent was evaporated under reduced pressure and the mixture was re-dissolved in DCM (100 mL), washed with water (2 × 100 mL) and brine (100 mL), dried over anhydrous $MgSO_4$, filtered and dried under reduced pressure. The crude product was then subjected to size exclusion chromatography (BioBeads, THF), where the intense brown fraction was collected. The solvent was removed under reduced pressure and the crude product was re-dissolved in THF, then passed through a compressed pad of celite. The solvent was removed under reduced pressure and the crude product was suspended in *n*-pentane,

thoroughly sonicated, filtered, washed with some more *n*-pentane, collected and dried in vacuum to yield **FePor(dmp)₂** (23.5 mg, 71%) as a dark-brown solid.

**$^{1}$H NMR** and **$^{13}$C NMR** were unsuccessful due to the paramagnetic nature of the inner $Fe^{II}$ metal ion. **APCI MS**: m/z 570.1716 - 575.1795 $[M+H]^{\bullet+}$; **HRMS** (APCI, positive): calcd for $C_{36}H_{28}FeN_4$ $[M]^{\bullet+}$: 572.1663; found: 572.1665; **UV/vis** ($CHCl_3$): $\lambda_{max}$ (log ε) = 371 (4.70), 406 (4.90), 499 (3.99), 574 (3.50), 639 (3.41); **FT-IR (ATR)** ν ($cm^{-1}$) = 3097, 2920, 2852, 1462, 1444, 1378, 1292, 1143, 1061, 995, 858, 791, 771, 732, 724.

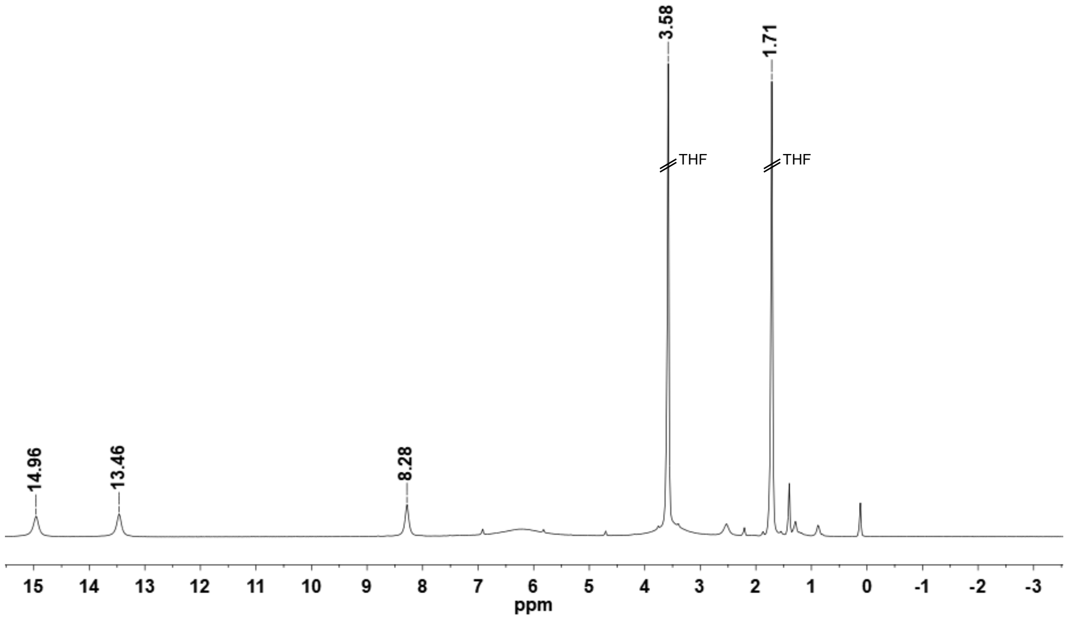


**Figure S3.11.** $^{1}$H-NMR spectrum of **FePor(dmp)₂** in THF-$d_8$. Due to the paramagnetic nature of the inner $Fe^{II}$ metal ion, only three (out of the expected 6) rather broad peaks were detected, while others are completely missing.

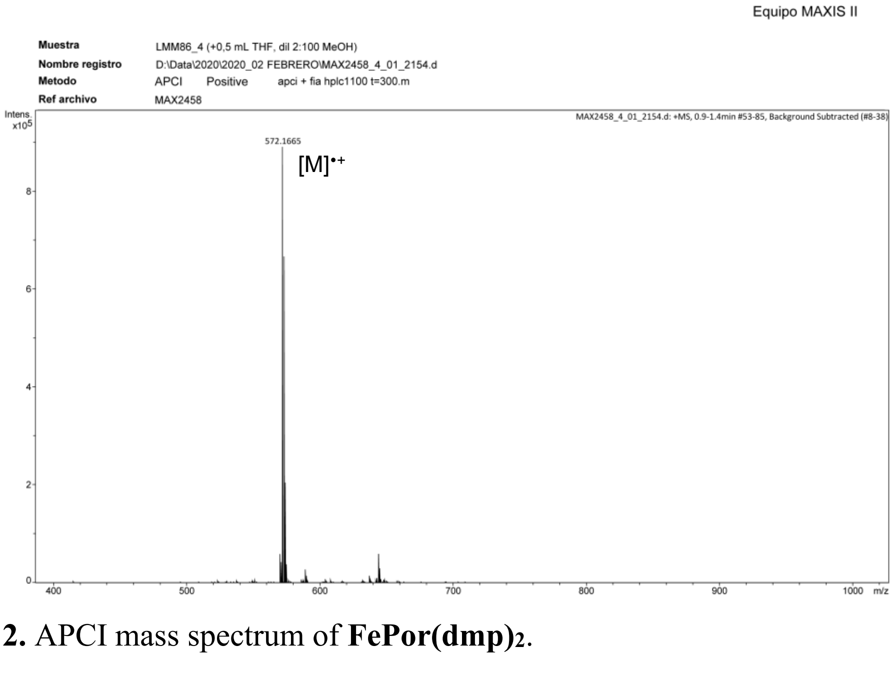


**Figure S3.12.** APCI mass spectrum of **FePor(dmp)₂**.

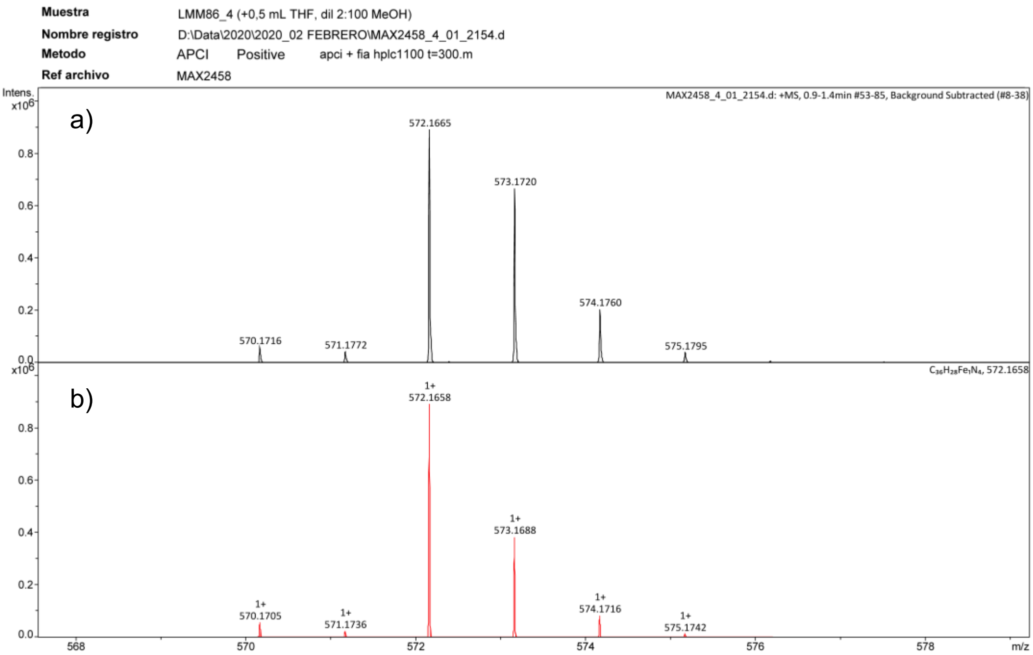


**Figure S3.13.** APCI HR mass spectrum of **FePor(dmp)₂**. a) Isotopic distribution of the APCI peaks between 570.1716 and 575.1795 m/z; b) calculated isotopic pattern for **FePor(dmp)₂**.

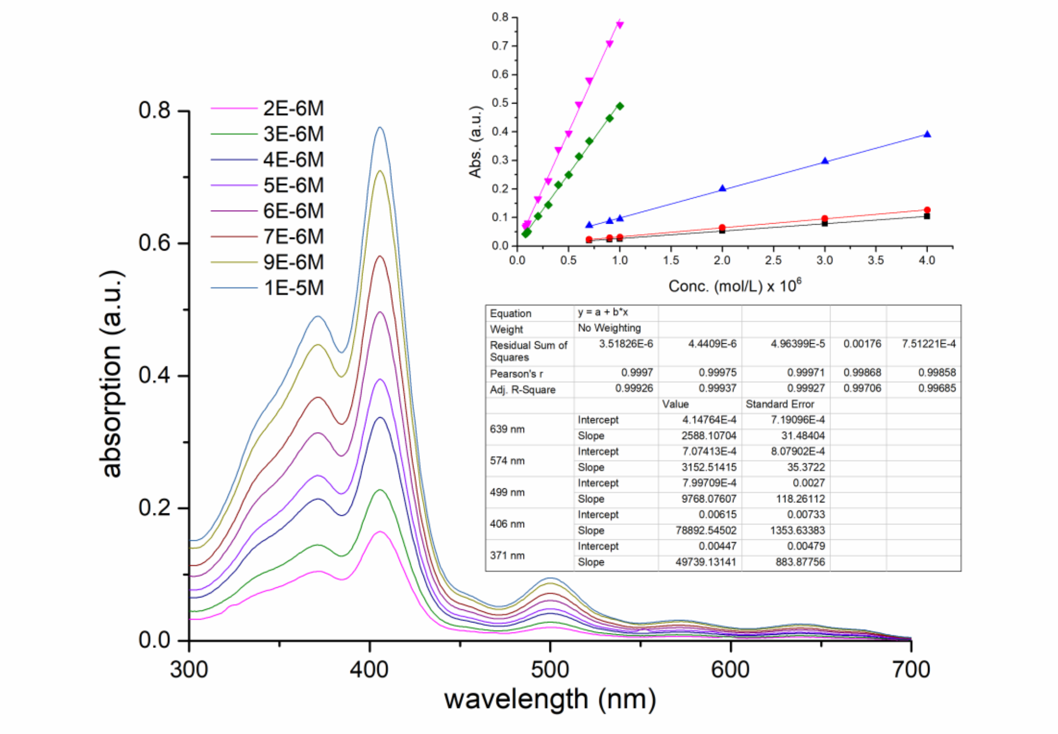


| | | | | | |
|---|---|---|---|---|---|
| Equation | y = a + b*x | | | | |
| Weight | No Weighting | | | | |
| Residual Sum of Squares | 3.51826E-6 | 4.4409E-6 | 4.96399E-5 | 0.00176 | 7.51221E-4 |
| Pearson's r | 0.9997 | 0.99975 | 0.99971 | 0.99868 | 0.99858 |
| Adj. R-Square | 0.99926 | 0.99937 | 0.99927 | 0.99706 | 0.99685 |
| | | Value | Standard Error | | |
| 639 nm | Intercept | 4.14764E-4 | 7.19096E-4 | | |
| | Slope | 2588.10704 | 31.48404 | | |
| 574 nm | Intercept | 7.07413E-4 | 8.07902E-4 | | |
| | Slope | 3152.51415 | 35.3722 | | |
| 499 nm | Intercept | 7.99709E-4 | 0.0027 | | |
| | Slope | 9768.07607 | 118.26112 | | |
| 406 nm | Intercept | 0.00615 | 0.00733 | | |
| | Slope | 78892.54502 | 1353.63383 | | |
| 371 nm | Intercept | 0.00447 | 0.00479 | | |
| | Slope | 49739.13141 | 883.87756 | | |

**Figure S3.14.** UV-Vis spectrum of **FePor(dmp)₂** in $CHCl_3$.

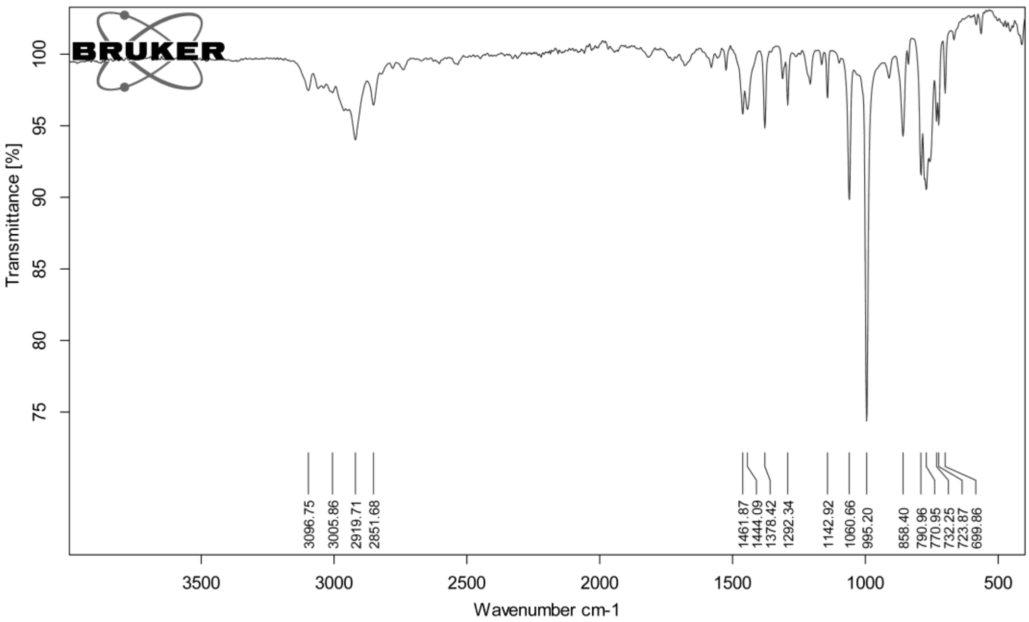


**Figure S3.15.** FT-IR spectrum of **FePor(dmp)$_2$**.

## 4. References

(1) Gross, L.; Mohn, F.; Moll, N.; Liljeroth, P.; Meyer, G., The chemical structure of a molecule resolved by atomic force microscopy, *Science* **2009**, *325*, 1110-1114.

(2) Giessibl, F. J., Atomic resolution on si(111)-(7×7) by noncontact atomic force microscopy with a force sensor based on a quartz tuning fork, *Appl. Phys. Lett.* **2000**, *76*, 1470-1472.
